# The Fractal Tapestry of Life: II Entailment of Fractional Oncology by Physiology Networks

**DOI:** 10.3389/fnetp.2022.845495

**Published:** 2022-03-24

**Authors:** Bruce J. West

**Affiliations:** ^1^ Center for Nonlinear Science, Univesity of North Texas, Denton, TX, United States; ^2^ Office for Research and Innovation, North Carolina State University, Rayleigh, NC, United States

**Keywords:** fractals, fractional calculus, oncology, networks, multifractals

## Abstract

This is an essay advocating the efficacy of using the (noninteger) fractional calculus (FC) for the modeling of complex dynamical systems, specifically those pertaining to biomedical phenomena in general and oncological phenomena in particular. Herein we describe how the integer calculus (IC) is often incapable of describing what were historically thought to be simple linear phenomena such as Newton’s law of cooling and Brownian motion. We demonstrate that even linear dynamical systems may be more accurately described by fractional rate equations (FREs) when the experimental datasets are inconsistent with models based on the IC. The Network Effect is introduced to explain how the collective dynamics of a complex network can transform a many-body noninear dynamical system modeled using the IC into a set of independent single-body fractional stochastic rate equations (FSREs). Note that this is not a mathematics paper, but rather a discussion focusing on the kinds of phenomena that have historically been approximately and improperly modeled using the IC and how a FC replacement of the model better explains the experimental results. This may be due to hidden effects that were not anticapated in the IC model, or to an effect that was acknowledged as possibly significant, but beyond the mathematical skills of the investigator to Incorporate into the original model. Whatever the reason we introduce the FRE used to describe mathematical oncology (MO) and review the quality of fit of such models to tumor growth data. The analytic results entailed in MO using ordinary diffusion as well as fractional diffusion are also briefly discussed. A connection is made between a time-dependent fractional-order derivative, technically called a distributed-order parameter, and the multifractality of time series, such that an observed multifractal time series can be modeled using a FRE with a distributed fractional-order derivative. This equivalence between multifractality and distributed fractional derivatives has not received the recognition in the applications literature we believe it warrants.

## Introduction

All phenomena are equally susceptible of being calculated, and all that is necessary, to reduce the whole of nature to laws similar to those which Newton discovered with the aid of the calculus, is to have a sufficient number of observations and a mathematics that is complex enough (Marquis de Cordorcet, 2022).

The modern science of medicine, like many other non-physical disciplines, has been guided in its early mathematical development by the successful mechanical models of physics. That strategy has proven to be extraordinarily successful, even surviving the introduction of fractals into its modeling, until quite recently. The true complexity of medical networks has been revealed with the development and implementation of ever more sensitive sensors and mathematically sophisticated data processing techniques (Niu et al., 2021). These developments have led to a divergence of the modeling strategies appropriate for the physical sciences from those for the life sciences.

Complex phenomena in any of the science disciplines have complicated and intricate behaviors, typically balancing randomness against order, with no consensus among scientists or poets as to what constitutes a reasonable scientific measure of complexity. Any list of traits of complexity is arbitrary and idiosyncratic and mine consists of eight traits which is recorded in *Where Medicine Went Wrong* (West, 2006). It would not serve our purpose to reproduce the details of that list here except to note that it contained such things as the number of time-dependent variables, along with the nonlinear relations among them, a dependence on their environment, scaling is space and time, and a mixture of order and randomness.

Recognizing the different ways each of these complexity properties are treated in the physical, social, and life sciences, led to further divergences of the modeling strategies developed for each. Therefore, we review how far the fractional calculus (FC) or alternatively fractional dynamics (FD) has taken us into the non-mechanical interpretation of medicine. On the one hand, network science has had a significant growth spurt over the last 2 decades with the recognition of its utility in describing the dynamic behavior of all manner of complex phenomena. This includes the recent establishing of new journal on Network Physiology (Ivanov, 2021). On the other hand, although the developers of network science have put such topics as scaling (Schmitt and Ivanov, 2007), renormalization group theory, fractal statistics (Bernaola-Galván et al., 2012), and other ostensibly esoteric mathematical tools into their bag of tricks, they have been slow to incorporate FD and the FC as a primary modeling strategy. Hopefully, the present essay will help to rectify that situation.

This essay is an unapologetic advocacy for the use of FC in the effective modeling of complex phenomena in biology and medicine. What emerges herein is the increasing importance of criticality (West, 2020), the cooperative nature of networks in healthy physiologic behavior (West et al., 2014), and the importance of the FC in characterizing the dynamics of living networks (West et al., 2008; West and Grigolini, 2021). In particular, we examine how and why the dynamic behavior of such pathologies as cancer may lend themselves to description by the FC (Nasrolahpour, 2018).

### Historical Perspective

The present paper is the sequel to *The Fractal Tapestry of Life: A review of Fractal Physiology* (West, 2021)*.* The prequel contains a critique of the reliance that physiology theory has had on the mechanical models of physics for its development, pointing out the extraordinarily success this strategy has enjoyed, see for example (Ruch and Patton, 1979). However, with the introduction of fractals by Mandelbrot (Mandelbrot, 1977) into the modeling strategy of science and engineering, the true complexity of physiological networks was revealed and led to the parting of the ways for the modeling strategies appropriate for the physical sciences from those for the life sciences. In the prequel we emphasized how far the fractal concept has taken us in the non-mechanical interpretation of physiology since the term *fractal physiology* was coined by Bassingthwaighte et al. (Bassingthwaighte et al., 1994) a quarter century ago. The prequel drew largely from papers published in the *frontiers in Physiology*, *Fractal Physiology* over my 2 decade tenure as its founding editor. The intent of that review was to demonstrate how far the modeling community has come in accepting fractals as a part of natural history.

As done in the prequel, the discussion presented herein draws inspiration for its rationale from Daniel Kahneman’s book, *Thinking, fast and slow* (Kahneman, 2011). Kahneman is a psychologist who was awarded the 2002 Noble Prize in Economics, suggesting that disciplinary borders, between economics and psychology in that case, are self-imposed barriers not supported by experiments done on the phenomena being studied. One consequence of the psychology experiments done and interpreted by him and his long time friend and collaborator Amos Tversky was that the historical assumptions about how humans make decisions, and in particular, how economic decisions on which microeconomics was based, had to be reexamined and some needed to be abandoned altogether. The assumption of strict rationality in humans, foundational to modern economic theory, turned out to be at odds with the empirical findings (Ariely, 2008).

The purpose of the present work is to demonstrate that we are now entering a new era in medicine, or rather in the mathematical modeling of medical phenomena, and what that entails for the future. It is useful to recall that in the *Principia* Newton introduced motion as a central idea of mechanics into physics, and although he never used the term *fluxion*, his word for a differential, in this major work he drew inspiration from his new mathematics to explore its implications. It was the mathematical notion of a differential that led Newton to identify motion as the central concept in celestial mechanics. He communicated this using the scientific language of the day, that being geometry, which explains some of the more torturous geometric arguments one finds in that remarkable book. His use of the differential historically guided the mechanics-based development of quantitative physiologic models, with some extraordinary successes, see, e.g., (Ruch and Patton, 1979) for a comprehensive discussion of the IC modeling of physiological systems.

On the other hand, this new era of medicine argues against relying on borrowing as a strategy for model building. It is not much of a stretch to say that typical phenomena in the life sciences are significantly more complex than those typically addressed in the physical sciences. Consequently, how one incorporates this complexity into the dynamic description of living cells, tissue and organs is uniquely defined by the phenomenon being considered (Magin, 2006; West et al., 2008). It is also the case that the way in which complexity enters the mathematical model determines the sensitivity of the model’s reaction to changes in parameter values. In other words the complexity of the phenomenon being modeled determines the degree of disruption that can be tolerated without the network degenerating into pathological behavior.

It is also the case that the standard training of the life scientist relies less on mathematical formalism, a reliance that enabled the success of the physical scientist in constructing useful physics-based mathematical models. However, scientists from both the physical and medical camps saw, almost immediately, the benefit of the fractal concepts for their respective domains of interest after it was introduced by Mandelbrot (Mandelbrot, 1977). But unlike Newton, who was working to understand a clearly observed physical phenomenon, Mandelbrot was attempting to understand not just the way we model change in the physical world, but change in every scientific discipline. To accomplish this ambitious goal he introduced the fractal concept through an endless succession of exemplars, including mathematical measures, noise, error, stellar matter, turbulence, statistics, polymers, and so on. These and many other applications of his ideas were based on his phenomenal intuition, using a kind of mathematics that neither the physical nor life sciences had seen before, much less implemented in the understanding of complex phenomena. Consequently, scientists in each discipline began developing fractal models based on what was needed to understand the unique processes and phenomena in their respective areas of study. It was equally clear that the fractal behavior of phenomena in living systems is the norm, and not the exception it seemed to be, at first, in the physical sciences (Mandelbrot, 1982).

In general, the complexity of a phenomenon molds the characteristics of the function used to describe its behavior. This is particularly true in describing how a function describing a phenomenon changes in time. The Weierstrass function, although expressed as an infinite Fourier series, does not have a finite integer derivative and was for that reason chosen by Richardson (Richardson, 1926) to describe the diffusion of a passive scalar in the turbulent flow of a fluid. It has been shown (Rocco and West, 1999) that such a function can have a finite fractional derivative, even when its integer derivative diverges. So what does this divergence property entail? It was determined during the last quarter century that an amazing number of familiar medical phenomena are described by fractal functions (West, 2006; West, 2021). Subsequently, it was argued that the equations of motion for such complex phenomena must be fractional, since a fractal function does not have integer-value derivatives and consequently cannot have Newtonian equations of motion (West, 2016). Thus, this essay is devoted to the whys and ways the FC enters into the dynamics of medicine and provides insight into certain medical pathologies including MO (Durrett, 2013).

## The Network Effect

The new millennium has witnessed the blossoming of two quite different strategies for the mathematical modeling of the complex dynamics of large collections of interacting elements that appear in medicine, those being network science (West et al., 2014; Barabasi, 2016; Newman, 2018) and the fractional calculus (Podlubny, 1999; West et al., 2003a; Magin, 2006). The adoption of the network science strategy for the study of complex phenomena such as epidemic spreading of diseases (Pastor-Satorras et al., 2015), neuronal avalanches (Hernandez-Urbina et al., 2016), and social dynamics (Bak, 1996; Castellano et al., 2009; Mahmoodi et al., 2017) is a consequence of the fact that these networks are composed of many simple, interconnected, and dynamically interacting elements (West, 2014). In a similar way, the popularity of the FC in research has grown in the modeling of processes characterized by long-term memory as well as spatial heterogeneity (Herrmann, 2011; West, 2016). This FC popularity stems from its particular mathematical formulation, based on various definitions of non-local differentiation and integration operators and its utility in describing the dynamics of fractal phenomena, both in space and time. Therefore, since the effects of spatial heterogeneity and memory are frequently observed in biological, social, and artificial networks (Magin, 2016; Meerschaert et al., 2017), the application of FC in the domain of complex networks is a natural step toward providing novel analytical tools that are capable of addressing research questions arising in the field of medicine, such as *fractional dynamics* (FD). For example, FD has been used to model the complex dynamics in biological tissue (Magin, 2010) and biomedicine (Nasrolahpour, 2017; Nasrolahpour, 2018), as well as in the growth of cancer cells (Valentim et al., 2021), the signal decay in MRIs (Magin, 2016), and finally in the bizarre statistical fluctuations in dilute suspensions of algae and bacteria (Zaid et al., 2011), to name a few applications that are subsequently discussed.

At the turn of the 20th century the foundation of biology started moving from the concept of homeostasis, which is compatible with the physical notion of regression to equilibrium, to the concept of homeodynamics, which involves periodicity (Lloyd et al., 2001; Tu and McKnight, 2006), chaos and complexity (Guzm´an et al., 2017). As far as the important biological role of periodicity is concerned, we invite the readers to consult the excellent review paper of Strogatz (Strogatz, 2000), which reveals a connection between homeodynamics and neurophysiology.

Despite how simple the basic elements of complex networks are assumed to be, such as in cooperative behavior of animals (Flack et al., 2018), in the flow of highway traffic (Bette et al., 2017), or in the cascades of load shedding on power grids (Yang et al., 2017), the network dynamics are invariably characterized by rich self-organized emergent behavior (West and Grigolini, 2021). However, in most cases solving a network of coupled nonlinear equations to describe the behavior of a network composed of *N* units is at best labor intensive and at worst it is intractable. Consequently, the primary focus of investigations into the behavior of complex networks has been on their global behavior (Dorogovtsev et al., 2008). This approach travels the path taken by classical statistical physics, starting from insights of Maxwell and Boltzmann that the description of the state of a gas or a solid could only be achieved over the scale of the entire system (Toda et al., 2004).

In the same way, the ability to portray the global behavior of a complex network is not free but comes at the price of not being able to quantify the Newtonian dynamics of the components of which it is composed. Typically, one attempts to infer the global dynamics by averaging the behavior of single elements within the system, following a bottom-up approach of mean field theory (Turalska and West, 2018). Turalska and West (Turalska and West, 2018) addressed the issue of depicting global dynamics by turning the question around and rather than joining the behaviors of single elements within the dynamic network, they asked whether it is possible to construct a description of the dynamics of the individual elements from information provided about the network’s global behavior. They approach the problem by considering statistical properties of the global variable.

Frequently, the macro, or coarse-grained, variables observed in complex networks display emergent properties of scale invariance in space and/or in time. This scale invariance is manifest by, for example, the inverse power law (IPL) scaling of waiting-time probability density functions (PDFs) that reveals the variability of the time intervals between events. These time intervals are manifest in heart rate variability (HRV), in stride interval variability (SIV) and breath interval variability (BIV), or in the occurrence of brainquakes (West and Grigolini, 2021). The IPLs that characterize the emergent macroscopic behavior are reminiscent of particle dynamics near a critical point, where a dynamic network undergoes a phase transition (Christensen and Moloney, 2005). However, despite the mathematical advances made by the renormalization group approach and self-organized criticality theories that have shown how scale-free phenomena emerge at critical points, the issue of determining how the emergent properties influence the micro-dynamics of individual units, such as the growth of a single cell within a network of cells, whether healthy or pathological, is still in its nascent phase.

### The Entailment of Network Dynamics

Grinstein et al. (1985) demonstrated that any discrete network, whose dynamics are defined in terms of local interactions, having symmetric transitions between states and random fluctuations originating from a thermal bath or internal dynamics is a member of the Ising universality class. One such dynamic complex network is given by the decision making model (DMM) (Bianco et al., 2008; Turalska et al., 2009; Turalska et al., 2011) and for clarity of discussion this is the dynamic model we implement in this section. Each individual unit of the DMM is a stochastic oscillator that statistically dithers between the two states, +1 and -1. The dynamics are modeled on a two-dimensional lattice and defined in terms of the probability of an individual at lattice point *i* to be in either state, by the coupled two-state master equation:

$$\frac{dp^{\left(i\right)}\left(t\right)}{dt}=G_{i}\left(t\right)p^{\left(i\right)}\left(t\right),$$ (1)

$$p^{\left(i\right)}\left(t\right)\equiv \left(\begin{array}{c} p_{1}^{\left(i\right)}\left(t\right)\\ p_{2}^{\left(i\right)}\left(t\right) \end{array}\right),$$ (2)

where $p^{\left(i\right)}\left(t\right)$ is the probability of the element *i* = 1, 2, …, *N* within the network at the time *t* is normalized such that $p_{1}^{\left(i\right)}\left(t\right)+p_{2}^{\left(i\right)}\left(t\right)=1$ for every *i* and changes with the fundamental transition rate *g*_0_ < 1 between states. The matrix of time-dependent coupling rates for individual *i* is given by:

$$G_{i}\left(t\right)=\left(\begin{array}{cc} -g_{12}^{\left(i\right)}\left(t\right) & g_{21}^{\left(i\right)}\left(t\right)\\ -g_{21}^{\left(i\right)}\left(t\right) & g_{22}^{\left(i\right)}\left(t\right) \end{array}\right);$$ (3)

where the individual transition rates are:

$$\begin{array}{ccc} g_{12}^{\left(i\right)}\left(t\right) & = & g_{0}\exp\left[-\frac{K}{M^{\left(i\right)}}\left(M_{1}^{\left(i\right)}\left(t\right)-M_{2}^{\left(i\right)}\left(t\right)\right)\right],\;\\ g_{21}^{\left(i\right)}\left(t\right) & = & g_{0}\exp\left[\frac{K}{M^{\left(i\right)}}\left(M_{1}^{\left(i\right)}\left(t\right)-M_{2}^{\left(i\right)}\left(t\right)\right)\right], \end{array}$$ (4)

and 0 ≤ *K* < *∞* is the strength of the interaction. On the regular two-dimensional lattice considered here the number of nearest neighbors is given by $M^{\left(i\right)}=4$ and $0\leq M_{1,2}^{\left(i\right)}\left(t\right)\leq 4$ denotes the count of nearest neighbors in states *s*_*i*_ (*K*, *t*) = ±1 at time *t* for every individual *i*. The probability that the single unit in isolation changes its state corresponds to the case of *K* = 0. When the coupling constant *K* > 0, a unit in state +1 (-1) makes a transition to the state -1 (+1) faster or slower according to whether $M_{1}^{\left(i\right)}\left(t\right)<M_{2}^{\left(i\right)}\left(t\right)$ or $M_{1}^{\left(i\right)}\left(t\right)>M_{2}^{\left(i\right)}\left(t\right)$ , respectively.

**FIGURE 1:**
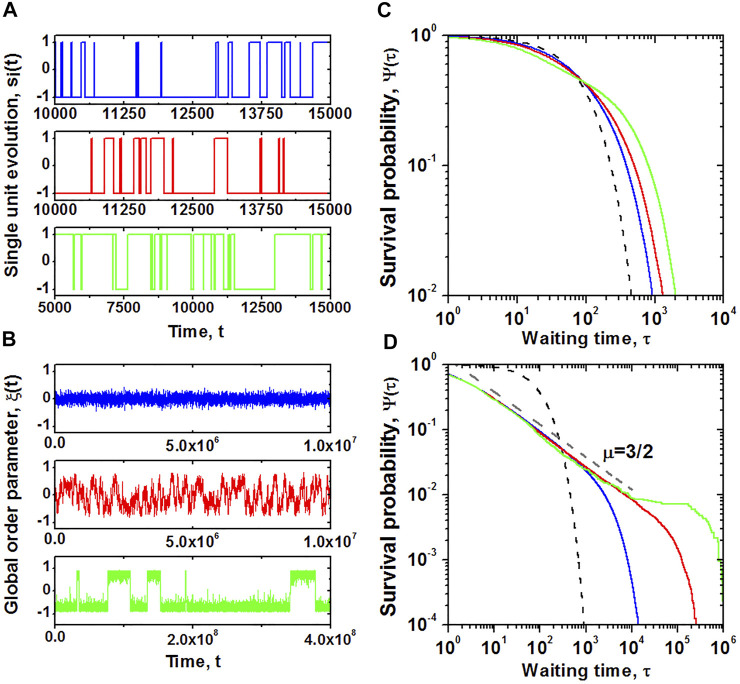
Behavior of a discrete, two-state dynamic unit on a two-dimensional lattice. Temporal evolution and corresponding survival probability $\Psi \left(\tau \right)$ for the transitions between two states for the single unit $s_{i}\left(t\right)$ of the network, presented on panels **(A,C)**, respectively, are compared with the behavior and statistical properties of the global order parameter $\xi \left(t\right)$ , shown on panels **(B,D)**. Simulations were performed on a lattice of size *N* = 50 × 50 nodes, with periodic boundary conditions, for *g*_0_ = 0.01 and increasing values of the control parameter *K*. Blue, red, and green lines correspond to *K* = 1.50, 1.70, and 1.90, respectively. The critical value of the control parameter is *K*_*C*_ ≈ 1.72. Black dashed line on the plots of $\Psi \left(\tau \right)$ denotes an exponential distribution, with the decay rate g_0_. From (Turalska and West, 2018) with permission.

This DMM network is defined by *N* such coupled equations, which gives rise to the problem of finding an analytic solution to a highly nonlinear network (Turalska et al., 2011) containing 2*N* dynamic variables. Given this number of dynamic variables extensive numerical calculations are supplemented by an analytic formulation of the evolution of a global variable. As depicted in Figure 1B, the global behavior of the model is defined by the fluctuations of the mean field variable:

$$\xi \left(K,t\right)=\frac{1}{N}\underset{i=1}{\overset{N}{\sum }}s_{i}\left(K,t\right),$$ (5)

which shows a pronounced transition as a function of the control parameter *K* as it passes through the critical value *K* = *K*_*c*_. The network dynamics for various quantities are depicted in the figure for the control parameter being subcritical (*K* < *K*_*c*_), critical (*K* = *K*_*c*_) and supercritical (*K* > *K*_*c*_). While in Figure 1A a typical single element appears to be essentially unchanged by its interactions with the rest of the network elements. On the other hand, the global variable shifts from a configuration dominated by randomness (subcritical) to one in which strong interactions give rise to long-lasting majority states (supercritical) shown in Figure 1B. Note that the source of the random fluctuation in the DMM is the finite size of the network, having a strength of $1/\sqrt{N}$ and has nothing to do with the thermal fluctuations arising in the modeling of phase transitions in physics phenomena such as the freezing of water or magnetization (West et al., 2014).

To characterize the changes in temporal properties of the network elements and those of the emergent properties of the macro variables, we evaluate the survival probability function $\Psi \left(\tau \right)$ , where *τ* is the time interval between consecutive events. These events are defined as changes of state or crossings of the zero-axis, for a single element or the global variable, respectively. These calculations reveal a modest deviation of the survival probability function for a single individual from the exponential form, $\Psi \left(\tau \right)=\exp\left(-g_{0}\tau \right)$ , that characterizes a single non-interacting element, as shown in Figure 1C. The network’s influence on the behavior of the individual appears to induce only a small change in the behavior of the latter. Despite this apparently small change in the individual’s behavior, the global variable changes dramatically, manifesting IPL statistics, as depicted in Figure 1D.

In this last panel three different aspects of dynamic behavior are revealed. The potential for the global variable is bimodal with the height of the barrier between the two potential minima determined by the parameter *K*. The dynamics are given by a Langevin equation with the strength of the random fluctuations driving the network from one well to the other decreasing with the size of the network as $1/\sqrt{N}$ . The subcritical and critical domains have dominant IPL survival probabilities trailing off into exponentials for long times. In the supercritical domain (green curve) a new phenomenon emerges called the *Kramers shoulder*. In his study of chemical reactions involving two states Kramers determined that the process becomes ergodic for times larger than what is now called the Kramers time, which increases exponentially with the size of the network, see West et al. (2014) for a detailed discussion.

Thus, to what extent are individual opinions within a complex network influenced by the network dynamics?

#### Complex Network Subordination

To determine the network’s influence on the dynamics of the individual unit we adapt a subordination argument, and relate the time scale of the macro variable *ξ*(*K*, *t*) to the time scale of the micro variable *s*_*i*_(*t*) following the arguments presented in (Turalska and West, 2018). The notion of different clocks associated with different aspects of a complex network dynamics dates back to the middle of the 19th century where the two clocks defined subjective and objective times and were used to justify the empirical Weber-Fechner law (Fechner, 1860). More recently, due to the availability of time resolved datasets, life science has begun adopting the notion of multiple clocks to distinguish between cell-specific and organ-specific clocks in biology, which is analogous to person-specific and group-specific clocks in sociology. While the global activity of an organ, such as the brain or the heart, might be characterized by quite regular, often periodic behaviors, the activity of single neurons or pacemaker cells demonstrate burstiness and noisiness. Thus, because of the stochastic behavior of the clocks, a transformation between clock times is necessary. An example of such a probabilistic transformation is the subordination procedure, see for example (Feller, 1966).

The two-state master equation for a DMM single isolated individual in discrete time *n* in steps of Δ*τ* can be written in terms of the single variable:

$$\varphi \left(n+1\right)-\varphi \left(n\right)=-g_{0}\Delta \tau \varphi \left(n\right)$$ (6)

in the notation $\varphi \left(n\right)=\varphi \left(n\Delta \tau \right)\;\equiv p_{1}\left(n\Delta \tau \right)-p_{2}\left(n\Delta \tau \right)$ being the difference in probabilities for the typical individual to assume one of two states. The solution to this discrete equation is after *n* ticks of the individual’s clock is:

$$\varphi \left(n\right)={\left(1-g_{0}\Delta \tau \right)}^{n}\varphi \left(0\right),$$ (7)

which, in the limit *g*_0_Δ*τ* ≪ 1, becomes an exponential. However, when the individual interacts with the other members of a network, the dynamics are no longer simple. Assuming a renewal property for events, an event being a transition from one state to the other, so that each event is independent of every other event, one can relate the discrete time of the unit to the clock time of the network using subordination theory.

Introducing subordination, we define the discrete index *n* as the *operational* time of the individual that is connected to the *chronological* time *t* recorded by the ticking of the network’s clock in which the global behavior is observed. If each tick of the discrete clock *n* is thought of as an event, then the relation between the operational time and the continuous chronological time can be given by the waiting-time PDF of those events in chronological time $\psi \left(t\right)$ . The chronological time lies in the interval (*n* − 1)Δ*τ* ≤ *t* ≤ *n*Δ*τ* for each operational tick and consequently the equation for the average dynamics of the individual probability difference is given by (Pramukkul et al., 2013):

$$\left\langle \varphi \left(t\right)\right\rangle =\underset{n=1}{\overset{\infty }{\sum }}\overset{t}{\underset{0}{\int }}\Psi \left(t-t^{\prime }\right)\psi_{n}\left(t^{\prime }\right)\varphi \left(n\right)dt^{\prime }.$$ (8)

Every tick of the operational clock is an event and occurs in chronological time at the drawing from the renewal waiting-time PDF *ψ*(*t*) determined by the derivative of the survival probability. The empirically determined analytic expression for the survival probability from the numerical simulation of DMM is:

$$\Psi \left(t\right)={\left(\frac{T}{T+t}\right)}^{\mu -1}e^{-\epsilon t}.$$ (9)

The dominant behavior of the empirical survival probability is IPL as indicated in Figure 1D. However, at early times the probability of not making a transition approaches the constant value of unity, whereas at late times the probability of not making a transition in a given time decays exponentially. It is in the middle range, where the survival probability is IPL. The extent of the IPL range of the survival probability is determined by the empirical values of *T*, *μ* and *ϵ* and from Figure 1D the value of *ϵ* is seen to become smaller as the control parameter *K* increases. The IPL functional form of the PDF results from the behavior of the survival probability $\Psi \left(\tau \right)$ of the global variable depicted in Figure 1D with *μ* = 3/2.

Using a renewal theory argument Pramukkul et al. (2013) show that Eq. 8 expressed in terms of Laplace transform variables indicated by $\hat{f}\left(u\right)$ for the time-dependent function *f*(*t*) has the form:

$$\left\langle \hat{\varphi }\left(s\right)\right\rangle =\frac{\varphi \left(0\right)}{u+\epsilon +\lambda_{0}\hat{\Phi }\left(u+\epsilon \right)}$$ (10)

where *λ*_0_ ≡ *g*_0_Δ*τ* and $\hat{\Phi }\left(u+\epsilon \right)$ is the Laplace transform of the Montroll-Weiss memory kernel (Pramukkul et al., 2013):

$$\hat{\Phi }\left(u+\epsilon \right)=\frac{\left(u+\epsilon \right)\hat{\psi }\left(u+\epsilon \right)}{1-\hat{\psi }\left(u+\epsilon \right)}.$$ (11)

Note that *u* is replaced by *u* + *ϵ* in the Laplace transforms, because the exponential truncation of the empirical survival probability shifts the index on the Laplace transform operation. The asymptotic behavior of an individual in time is determined by considering the waiting-time PDF as *u* + *ϵ* → 0:

$$\hat{\psi }\left(u+\epsilon \right)\approx 1-\Gamma \left(1-\alpha \right)T^{\alpha }{\left(u+\epsilon \right)}^{\alpha }\text{ ; }0<\alpha =\mu -1<1,$$ (12)

so that Eq. 10 reduces to:

$$\left\langle \hat{\varphi }\left(u\right)\right\rangle =\frac{\varphi \left(0\right)}{u+\epsilon +\lambda^{\alpha }{\left(u+\epsilon \right)}^{1-\alpha }}.$$ (13)

The inverse Laplace transform of Eq. 13 yields the tempered non-integer rate equation:

$${\left(\partial_{t}+\epsilon \right)}^{\alpha }\left\langle \varphi \left(t\right)\right\rangle =-\lambda^{\alpha }\left\langle \varphi \left(t\right)\right\rangle ,$$ (14)

where the operator $\partial_{t}^{\mu -1}\left[\cdot \right]$ is the Caputo fractional derivative For the moment we define the Caputo fractional derivative in terms of its Laplace transform:

$$\mathrm{LT}\left[\partial_{t}^{\alpha }\left[f\left(t\right)\right];u\right]=u^{\alpha }\hat{f}\left(u\right)-u^{\alpha -1}f\left(0\right),$$

where *f* (0) is the initial value of *f*(*t*) and $\hat{f}\left(u\right)$ is its Laplace transform for 0 < *α* = *μ* − 1 < 1 (West, 2016) and:

$$\lambda T={\left[g_{0}\Delta \tau /\Gamma \left(2-\mu \right)\right]}^{\frac{1}{\mu -1}}.$$ (15)

Note that due to the dichotomous nature of the states that $\left\langle \varphi \left(t\right)\right\rangle$ is the average opinion of the individual *s*_*i*_(*t*).

A technique for obtaining the solution of the asymptotic fractional master equation Eq. 14 is given in the following section in some detail. For the moment we solve the equation for a randomly chosen unit within the social network and obtain an exponentially attenuated Mittag–Leffler function (MLF) (Turalska and West, 2018):

$$\left\langle \varphi \left(t\right)\right\rangle =\varphi \left(0\right)E_{\alpha }\left(-{\left(\lambda t\right)}^{\alpha }\right)\exp\left[-\epsilon t\right].$$ (16)

and the MLF is defined by the series:

$$E_{\alpha }\left(z\right)\equiv \overset{\infty }{\underset{n=0}{\sum }}\frac{z^{n}}{\Gamma \left(n\alpha +1\right)},\;\alpha >0.$$ (17)

The MLF is a stretched exponential at early times and an IPL at late times, with *α* = *μ* − 1 being the IPL index in both domains (West et al., 2003a). The MLF will be discussed more fully in subsequent sections.

#### Comparisons With Numerics

**FIGURE 2:**
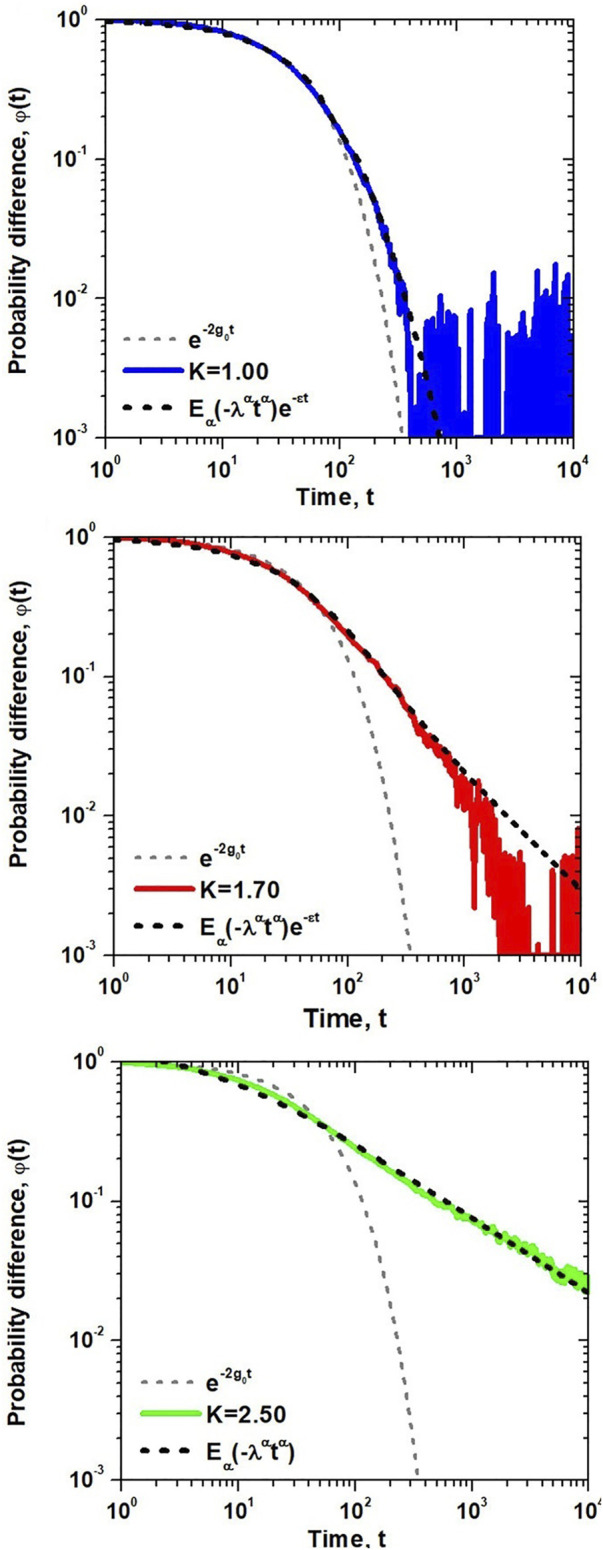
The probability difference $\left\langle \varphi \left(t\right)\right\rangle$ estimated as an average over an ensemble of 10^4^ independent realizations of single element trajetories. Each trajectory corresponds to evolution of a randomly selected node within a *N* = 100 × 100 lattice network, with *g*_0_ = 0.01 and the same initial condition *s*_*i*_ (0) = 1. The parameter values for the numerical data are given in Figure 1 and from top to bttom *K* = 1.0, 1.7, 2.5, respectively. The fit of the exponentially truncated MLF to the numerical calculations over the time interval [a,b] yields the parameter values: *K* = 1.0, *ϵ* = 4 × 10^−3^, *λ* = 1.47 × 10^−2^, *α* = 0.892 [1, 300]; *K* = 1.70, *ϵ* = 1.4 × 10^−11^, *λ* = 2.06 × 10^−2^, *α* = 0.805, [1, 10^3^]; *K* = 2.50, *ϵ* = 5.58 × 10^−12^, *λ* = 2.93 × 10^−2^, *α* = 0.558 [1, 10^4^]. Adapted from (Turalska and West, 2018) with permission.

We test the above solution against the numerical simulations of the dynamic network consisting of *N* = 10^4^ units on a two-dimensional lattice with nearest-neighbor interactions in all three regions of DMM dynamics; subcritical, critical and supercritical. The time-dependent average opinion of a randomly chosen individual is presented in Figure 2, where the average is taken over 10^4^ independent realizations of the dynamics in the three regimes.

**FIGURE 3:**
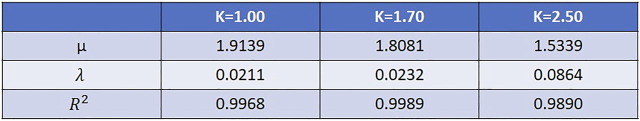
The probability difference $\left\langle \varphi \left(t\right)\right\rangle$ of Figure 2 is fitted with the MLF using an algorithm developed by Podlubny (Gorenflo et al., 2002). Assuming *T* = 0.10, Δ*τ* = 1 and *g*_0_ = 0.01 the parameters of analytic solution are *μ* = 3/2 and *λ* = 0.031 8. The mean-square goodness of fit *R* ^2^ is discussed in (Turalska and West, 2018).

A comparison with the exponential form of $\left\langle \varphi \left(t\right)\right\rangle$ for an isolated individual depicted by the dashed line segment depicted in Figure 2 indicates that the influence of the network on the individual’s dynamics clearly persists for increasingly longer times with increasing values of the control parameter within the network. The parameters *μ* and *λ* of Eq. 16 obtained through fitting numerical results of Figure 2 with the MLF are summarized in the table given in Figure 3. It is evident that the influence of the network dynamics on the individual is greatest at long times. The deviation of the analytic solution to the FRE from the numerical calculation is evident for values of the control parameter at and below the critical value. The analytic prediction is least reliable at extremely long times in the subcritical domain. Consequently, the response of the individual to the group, mimics the group’s behavior most closely when the control parameter is equal to or greater than the critical value.

### Complex Networks Entail Fractional Space Diffusion Equations

Herein the subordination procedure provides an equivalent description of the average dynamics of a single individual within a complex network in terms of a linear fractional stochastic rate equation (FSRE). The fractional dynamics given by Eq. 14 is solved exactly, determining that the Poisson statistics of the isolated individual, becomes attenuated Mittag–Leffler statistics, due to the interaction of that individual with the other members of the complex dynamic network. The numerical simulation of the network dynamics consisting of ten thousand nonlinearly interacting units collapses onto a one-dimensional fractional dissipative rate equation for a typical single unit. Note that the average influence of the other 9,999 units in the nonlinear dynamic network on the unit of interest is predicted by the MLF solution of the linear FRE without linearizing the dynamics. Let me say this again *the FRE is an exact representation of the complete response of a typical unit to the rest of the nonlinear dynamic network without linearizing the dynamics.* The effect of the other 9,999 units on the typical one is two-fold giving rise to an attenuated MLF dynamics with an attenuation rate *ɛ* and the noninteger-order derivative *α*, apparently without approximation.

As Pointed out in West (West, 2016): The results in this section provide a partial answer to a question in sociophysics identified by Kulakolwski and Nawojczyk (Kulakolwski and Nawojczyk, 2008) concerning how empirical regularities such as prejudice or tolerance can be derived from global social properties such as entropy or temperature. We can interpret their use of the nomenclature “temperature” as the control parameter in the DMM network. Here again we have demonstrated how the state of the network, as described by the global dynamics, can influence the decision making behavior of the individual.

This quote can be recast in the form where sociophysics is replaced by medical biophysics and the same partial answers can be obtained for how the empirical regularities such that the ubiquity of IPL statistics can be derived from global properties of physiological networks. We shall pursue this more fully in subsequent sections.

We conjecture that the behavior of the individual units are generic, given that the DMM network dynamics belong to the Ising universality class. Members of this universality class share the critical temporal behavior (West et al., 2014) driving the subordination process. It is the renewal property of the event statistics, which through the subordination process, gives rise to the linear fractional master equation for the typical unit’s dynamics. The solution to the tempered FRE manifests a subsequent robust behavior of the individual. It remains to determine just how robust the behavior of the individual is relative to control signals that might be used to manage healthy dynamics, as well as any pathologies that arises in the dynamics over the lifetime of the living network.

Thus, a unit’s simple random behavior, when isolated, is replaced with behavior that could serve a more adaptive role in social and medical networks. Think of the difference in the dynamics of an isolated pacemaker cell and that of the sinus node, the heart’s natural pacemaker. One might consider the solution to the following FSRE:

$${\left(\partial_{t}+\epsilon \right)}^{\alpha }\xi \left(t\right)=-\lambda^{\alpha }\xi \left(t\right)+V\left(t\right),$$ (18)

and *V*(*t*) represents parasympathetic and sympathetic fluctuating signals from the autonomic nervous system and *ξ*(*t*) is the mean field electrical output of the sinus node. The two branches of the nervous system are in an on-going tug-of-war in driving the sinus node, one decreasing and the other increasing the heart’s rate thereby producing the HRV time series in healthy subjects.

We close this section with the observation that the aggregate effect of the network dynamics is to reduce the 2*N* − dimensional master equation description of the nonlinear evolution of the probability to a 1 − dimensional description of the linear fractional dynamics of the global variable. Therefore, Eq. 18 is a generic representation of such dynamics with the formal solution in Laplace space:

$$\hat{\xi }\left(u\right)=\frac{{\left(u+\epsilon \right)}^{\alpha -1}\xi \left(0\right)}{{\left(u+\epsilon \right)}^{\alpha }+\lambda^{\alpha }}+\frac{\hat{V}\left(u\right)}{{\left(u+\epsilon \right)}^{\alpha }+\lambda^{\alpha }},$$ (19)

where the homogeneous solution $\xi_{h}\left(t\right)$ is obtained from Eq. 16:

$$\xi_{h}\left(t\right)=\xi_{h}\left(0\right)E_{\alpha }\left(-{\left(\lambda t\right)}^{\alpha }\right)\exp\left[-\epsilon t\right],$$ (20)

and the complete time-dependent solution is obtained by Laplace inversion to be (West et al., 2003a):

$$\xi \left(t\right)=\xi_{h}\left(t\right)+\overset{t}{\underset{0}{\int }}dt^{\prime }{\left(t-t^{\prime }\right)}^{\alpha -1}E_{\alpha ,\alpha }\left(-{\left[\lambda \left(t-t^{\prime }\right)\right]}^{\alpha }\right)\exp\left[-\epsilon \left(t-t^{\prime }\right)\right]V\left(t^{\prime }\right),$$ (21)

where we have introduced the generalized MLF:

$$E_{\alpha ,\beta }\left(z\right)\equiv \overset{\infty }{\underset{n=0}{\sum }}\frac{z^{n}}{\Gamma \left(n\alpha +\beta \right)},\;\alpha ,\beta >0.$$ (22)

The formalism represented here in the solution given by Eq. 21 is probably overwhelming if you are seeing it for the first time. So for the sake of clarity let us take a step back and systematically prepare the ground for solving differential equations involving noninteger operators before interpreting the above solution. The take away message from introducing such FDEs is that the solution to a ten thousand component master equation using IDEs has been expressed as a global variable solution to an appropriate FDE. As a medical application consider a tissue consisting of a large number of cells and what a practitioner could do with a model that exploited such a startling mathematical simplification.

## Applications of Fractional Calculus

Since the time of Newton science has accepted the explanation that the myriad kinds of motions of the objects in the physical world around us are determined by energy. Electrical energy provides the power that runs social media, the internet and lights our cities; chemical energy supplies the power to drive the engines in our transportation systems, and solar energy is converted by photosynthesis into the foods we eat. Physics provides a detailed description of how changes in energy over spatial intervals produce forces, which moves things around. A kite pulling at its tether, the invariant order of the colors in a rainbow, moon rise, and sunset, all have their causal explanations in terms of forces. But the force laws, even when generalized to continuous media such as fluids, are not able to explain everything. We talk of individuals exerting forces on one another, of stress in a relationship or in the work place. Is the latter force merely an analogue of the mechanical forces and thereby lacking material substance, or is it something more? Or are the dynamics of living networks really no different from those for inanimate matter?

We do not have complete answers to such profound questions, but what we can say is that models using IC from theoretical physics when applied to the complex phenomena of social and life sciences have, by and large, been disappointing. Here we argue that much of the disappointment encountered in the development and application of models outside the physical sciences has been the result of the simplifying assumptions made. Very often the simplifying assumption were known to be wrong but were necessary to satisfy the known properties of the mathematics used to construct the models. Other times the assumptions were idealizations that although not entirely accurate, were thought to capture the dominant characteristic of the phenomenon being investigated, and therefore the idealized model was wrong, but conveyed the truth. This is not unlike the children stories in *Aesop’s Fables* that teach abstract lessons in ethics and morality in a language children can understand.

The purpose here is not to belittle the mathematical techniques used in the past to understand the unifying nature of physical laws, but rather to highlight the fact that the only way we can formulate questions is by means of language and mathematics is the language of science: for Galileo the language was geometry and algebra; for Newton and scientists for the following three centuries the language was primarily the differential calculus. Consequently, much of what is presented is concerned with the mental map of the world we construct from such mathematics. An exhaustive treatment of the social implications of the limit concept has been treated by Amir Alexander in his remarkable book *Infinitesimal, How a Dangerous Mathematical Theory Shaped the Modern World* (Alexander, 2014):

On 10 August 1,632, five men in flowing black robes convened in a somber Roman palazzo to pass judgment on a simple proposition: that a continuous line is composed of distinct and infinitely tiny parts. The doctrine would become the foundation of calculus, but on that fateful day the Jesuit fathers ruled that it was forbidden. With the stroke of a pen they launched a war for the very soul of the modern world.

This dramatic depiction of the dispute over a mathematical concept lay at the heart of what was the Catholic Church’s role in interpreting how we humans were to understand the world in which we live. It is not my purpose here to present Alexander’s brilliant historical arguments on how the concepts of continuity and limit became a fundamental theological issue. Instead I wish to emphasize that then, as now, our understanding of the world is based on the language we use to describe it and which necessarily determines how we can think about it. Our mental models of the world and its events are all we have, so when we embark on scientific investigations it is in our fundamental interest to refine those models as best we can.

This is where the FC enters the discussion. It is not surprising that colleagues should ask about noninteger differentials and integrals and they did so in Newton’s lifetime, asking Leibniz the cofounder of the differential calculus if such noninteger operators could be defined. Other than these technical questions addressed in private letters the noninteger aspect of the calculus was mostly ignored by the social, physical, and life scientists, intermittently emerging from the shadows of formalism with an application over the centuries. The international scientific community saw no need for a new calculus. As a body, the science community tacitly agreed that the ordinary differential calculus, along with the analytic functions entailed by solving the equations resulting from Newton’s force law, are all that was required to provide a scientific description of the macroscopic physical world.

As pointed out elsewhere (West, 2020) the evidence is all around us that the domain of application of Newton’s view of the physical world is contracting dramatically. This is one result of the increase in sensitivity of diagnostic tools, advances in data processing techniques, and expanded computational capabilities, which have all contributed to the broadening of science in ways that have pushed many phenomena from borderline interest to center stage. These curious complex processes are now catalogued under the heading of fractal scaling phenomena and their impact has nowhere been more emphatic than in medical science (West, 2016). The understanding of the fundamental dynamics underlying such scaling requires the new mathematical perspective obtained using fractional operators and such descriptions have apparently ushered in the sunset for much of what remains of Newton’s world view.

### Fractional Differential Equations

FC is concerned with the quantitative analysis of functions using non-integer-order operators that generalize the traditional meaning of integration and differentiation. The non-integer order of a FC operator can be a real number, a complex number, or even the function of a variable, as we shall see. But this is less an essay on mathematics, than it is a presentation of the remarkable scientific utility of the FC, whose scope is intended here for medical scientists who recognize the need for such methods but may be less interested in learning the formal details of the methodology. So let us begin by examining well-accepted, simple, linear, dynamic processes that turn out to be not so simple.

#### Newtonian Cooling

We begin with an example presented by Mondol et al. (Mondol et al., 2018) who used a FDE to examine Newton’s linear cooling law. This law states that the rate of heat loss from a body is directly proportional to the difference in the temperatures between that of the body at time *t* denoted as *T*(*t*) and that of its surroundings *T*_*a*_ given by the IRE:

$$\frac{dT\left(t\right)}{dt}=-\lambda \left[T\left(t\right)-T_{a}\right],$$ (23)

with loss rate *λ*, provided the temperature difference is not too large and the nature of the radiating surface remains the same throughout the time of the cooling process. The prediction of this law is that cooling proceeds at a constant rate from the initial temperature of the object *T*_0_ to the ambient temperature *T*_*a*_ and is exponential in time:

$$T\left(t\right)=T_{0}e^{-\lambda t}+T_{a}\left(1-e^{-\lambda t}\right),$$ (24)

since it is the solution to a linear rate equation. This solution allows the medical examiner to unambiguously establish the time of death in every television murder mystery ever made. However, in the real world Mondol et al. determined experimentally that the cooling problem is not so simple and does in fact depend more subtilely on the properties of the objects being cooled.

**FIGURE 4:**
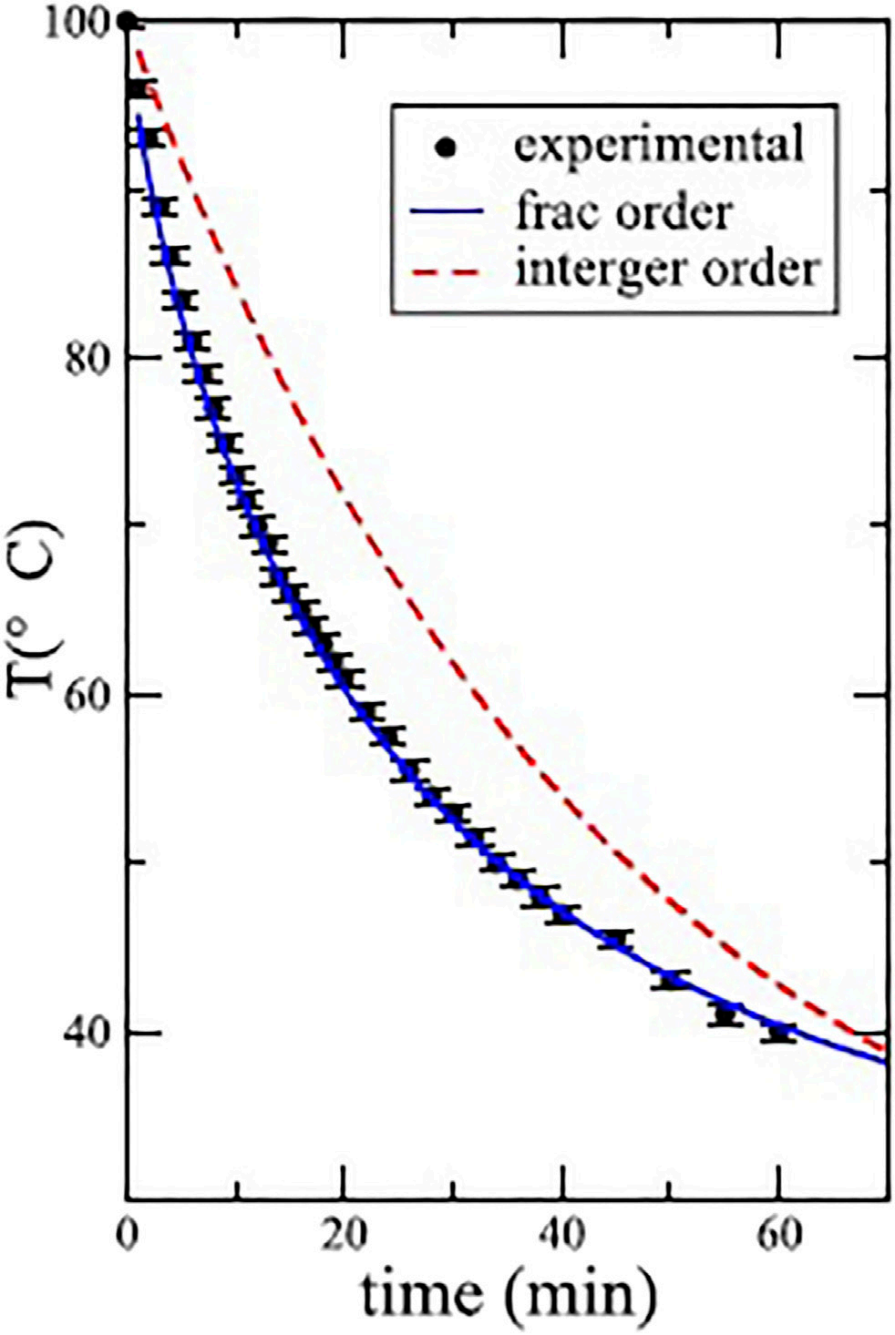
Comparison of the experimental cooling data with solutions using the Caputo derivative in Eq. 25 with *α* = 0.79 and the exponential for 300 ml of water. Adapted from Mondol et al. (2018) with permission.

In general, they replace Newton’s cooling equation with the FRE:

$$\partial_{t}^{\alpha }\left[T\left(t\right)\right]=-\lambda \left[T\left(t\right)-T_{a}\right],$$ (25)

where again the ambient temperature is *T*_*a*_. Notice also that the solution to cooling equation given by Eq. 25 using a Caputo noninteger derivative $\partial_{t}^{\alpha }\left[\cdot \right]$ has the same form as Eq. 24 with the exponential replaced with a MLF:

$$T\left(t\right)=T_{0}E_{\alpha }\left(-\lambda t^{\alpha }\right)+T_{a}\left[1-E_{\alpha }\left(-\lambda t^{\alpha }\right)\right],$$ (26)

a complete discussion of this solution is given elsewhere (West, 2016; Mondol et al., 2018). In Figure 4 the dashed curve depicts the “best” exponential solution to Newton’s cooling law, which clearly deviates from the experimental data given for 300 ml of water. The solution to the FRE is given by Eq. 26 and is depicted by the solid curve, fitting the experimental data extremely well.

Of course, this is just one example selected from the many experimental results Mondol et al. present. These IRE and FRE predictions compared to the experimental datasets emphasize the error one can make in modeling even familiar linear dynamic phenomena that one has no reason to believe are not simple. But this is a cautionary tale. One should consider every phenomenon to be complex or nonsimple until it is verified by both experiment and theory to be simple. In the case of an object cooling over time the nonsimplicity has to do with memory that is built into the definition of the FC derivatives irrespective of their detailed forms. Mondol et al. (Mondol et al., 2018) demonstrate experimentally that some cooling phenomena are represented by Newton’s law of cooling, others by the Caputo noninteger form as in the example shown in Figure 4 and still others by the Reiman-Liouville form of the FC derivative operator, both of which will be defined in due course. The need for the fractional (noninteger) differential is explained in the following manner (Mondol et al., 2018):

Thus, it is all about taking rate of change of a variable…with respect to fractional differential of time which defines Δ*t*, the “window of observation.” While the classical differential is Δ*t* ^*α*^ with *α* = 1, the fractional differential is Δ*t* ^*α*^ with *α* < 1…(thus) the fractional differential will always be greater than classical differential as Δ*t* → 0. This makes the “window of observation” to view complex dynamics effectively larger, as compared to Δ*t* …as we make Δ*t* go from milli, to micro, to pico…the fractional differential …grows, helping us to view the dynamics which may be complex with several relaxation processes and relaxation rates, better.

They go on to say that if the dynamics is governed by a unique cooling rate, there is no need to increase the viewing window by using a fractional (noninteger) differential and Newton’s cooling law prevails. Thus, if you are convinced that the process you are dealing with is linear, but the data deviate systematically from any linear IDE model you have examined, the deviation may be the result of non-locality and not a nonlinearity. The questions you need to answer is which calculus Nature has chosen for the dynamics of the phenomena under investigation and why?

#### Brownian Motion

The only exposure to stochastic processes that typically resonates with non-mathematically oriented scientists is the phenomenon of Brownian motion. The phenomenon was observed by the botonist Robert Brown in 1827, who studied the erratic motion of a pollen mote suspended in a fluid using a microscopic. He hoped that his observation would explain the ‘life force’ he thought at first he was observing, which he admittedly did not accomplish. Of course, it turned out that he was watching the reaction of the pollen mote to the thermal motion of the invisible molecules of the ambient fluid as explained by Einstein in his 1905 paper on diffusion (Einstein, 1905). In a 1907 sequel to this paper Einstein speculated, after informally hearing of these early experiments, that Brown could well have been observing a diffusive process (Einstein, 1907). This off-hand remark in a published paper was sufficient to insure Brown’s scientific immortality.

Einstein did however recognize a problem with his formulation of what is now known as Brownian motion. If *X*(*t*) is the instantaneous position of a free Brownian particle its mean-square displacement (MSD) from its initial position is predicted by Einstein’s theory to be *X*(0):

$$\left\langle \Delta X{\left(t\right)}^{2}\right\rangle =\left\langle {\left[X\left(t\right)-X\left(0\right)\right]}^{2}\right\rangle =2Dt,$$ (27)

where *D* is the diffusion coefficient and *t* is the time. Without going into the underling physics of molecular diffusion we can observe, as did Einstein, that the average velocity $\bar{V}$ over a time *t* can be estimated using the MSD:

$$\bar{V}=\frac{\bar{\Delta X}}{\Delta t}=\frac{\sqrt{\left\langle \Delta X{\left(t\right)}^{2}\right\rangle }}{t}=\frac{\sqrt{2D}}{\sqrt{t}}.$$ (28)

Consequently, for very short times, where one might expect the estimate to be better, the mean velocity diverges to infinity and therefore cannot represent a real velocity (Einstein, 1907). Using an argument based on the equipartition theorem of statistical physics Einstein concluded (Einstein, 1907):

We must conclude that the velocity and direction of motion of the particle will be already very greatly altered in the extraordinary short time *θ*, and, indeed, in a totally irregular manner. It is therefore impossible - at least for ultramicroscopic particles - to ascertain $\sqrt{\bar{V^{2}}}$ by observation.

As Li and Raizen (2013) point out, it took more than a century for Einstein’s conclusions to be experimentally challenged because of the technical difficulties of doing an experiment that can resolve a Brownian particle at times on the order of nanoseconds and within distances on the order of the radius of a hydrogen atom. Li et al. (2010) were able to achieve the incredibly high resolution in space and time necessary to measure the ballistic motion of the Brownian particle between molecular collisions using optical trapping interferometry.

Langevin’s theory of Brownian motion (Langevin, 1908), based as it is on dynamic equations and not on probability arguments, is ostensibly valid at all times including for times much smaller than that for diffusion of the Brownian particle. It predicts ballistic motion for such short times and as a consequence the MSD is independent of the diffusion coefficient *D* and is given directly in terms of the fluid temperature *T*, Boltzmann’s coefficient *k*_*B*_, and the mass of the Brownian particle *M*:

$$\left\langle \Delta X{\left(t\right)}^{2}\right\rangle =\frac{k_{B}T}{M}t^{2},$$ (29)

yielding the average velocity:

$$\bar{V}=\frac{\sqrt{\left\langle \Delta X{\left(t\right)}^{2}\right\rangle }}{t}=\sqrt{\frac{k_{B}T}{M}},$$ (30)

a well-defined constant.

**FIGURE 5:**
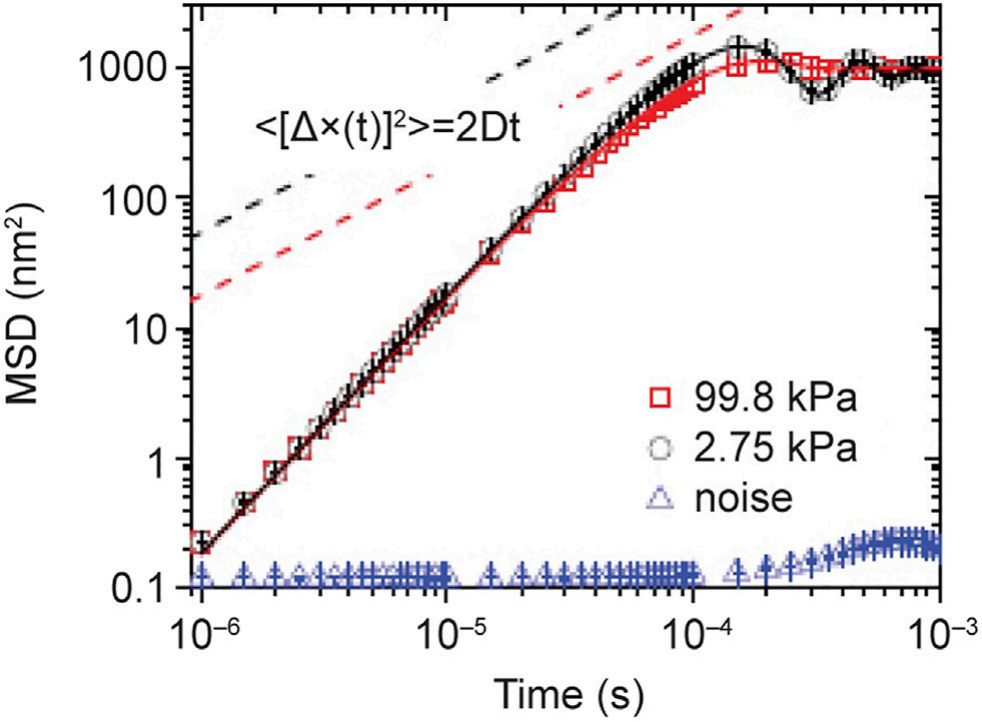
The MSDs of a 3 *μm* silica bead trapped in air at 99.98 *kPa* (red squares) and 2.75 *kPa* (black circle). They are calculated from 4 × 10^7^ measurements for each pressure. The “noise” signal (blue triangles) is recorded when there is no particle in the optical trap. The solid lines are the theoretical predictions of Eq. 31. The prediction of Eistein’s theory of free Brownian motion in the diffusive regime is shown in dashed lines for comparison. From (West, 2016) with permission originally published by (Li and Raizen, 2013).

Li et al. (2010) determined the position and velocity of a 3 *μm* diameter silicon sphere confined in an air optical trap configured in a vacuum chamber. Without presenting the details of the experiment it is sufficient to note that the optical trap harmonically confines the particle in physical space where it is subject to thermal collisions with the air particles in the chamber. It is evident from Figure 5 that the measured MSD deviates markedly from Einstein’s theory of Brownian motion in the early time regime.

Langevin theory gives the proper ballistic behavior for a harmonic oscillator driven by random noise and the MSD was originally obtained in 1930 by Uhlenbeck and Ornstein (Uhlenbeck and Ornstein, 1930):

$$\left\langle {\left[X\left(t\right)-\left\langle X\left(t\right)\right\rangle \right]}^{2}\right\rangle =\frac{2k_{B}T}{M\omega_{0}^{2}}\left\{1-e^{-t/\tau_{D}}\left[\cos\omega_{1}t+\frac{\sin\omega_{1}t}{2\omega_{1}\tau_{D}}\right]\right\}$$ (31)

where *τ*_*D*_ is the diffusion time scale, *ω*_0_ is the resonant frequency of the optical trap and $\omega_{1}=\sqrt{\omega_{0}^{2}-{\left(1/2\tau_{D}\right)}^{2}}$ . Note that the strength of the fluctuations driving the Langevin equation in the experiment are not proportional to the diffusion coefficient but to the temperature of the ambient air in the chamber. This solution is given by the solid curve in Figure 5 with the parameter values determined from the experiment the fit to the data is excellent.

Like the story of Newton’s linear theory of cooling the present tale of Brownian motion does not end here. You may have noticed that Einstein’s theory was based on a Brownian particle in water, whereas Langevin’s theory is applied to one in air and this highlighted the difference between the two theories at short times. The properties of the ambient fluid turn out to make a tremendous difference in which theory to apply because the momentum relaxation time scale in a liquid phase is 50 times greater than in a gas phase due to the difference in the ambient fluid density. It turns out that neither Einstein nor Langevin got it entirely right, because the dynamics of a freely moving Brownian particle is more subtle than either of them imagined. Brownian motion had been separated into microscopic and macroscopic by both men but the phenomenon actually lives in the in-between world of the mesoscopic.

The force equation used to describe Brownian motion by Langevin is given by the direct application of Newton’s Third Law to a spherical particle in water and that turned out to be the wrong approach. The phenomenon is more subtle than that. It requires taking into account the inertia of the ambient fluid. The derivation of the equation for the motion of a heavy spherical particle in a fluid, requires taking into account the back-reaction of the ambient fluid in contact with the Brownian particle. This back-flow of the fluid was first derived in 1885 by Boussinesq (Boussinesq, 1885) and independently 3 years later by Basset (Basset, 1888).

For a spherical particle of radius *R* in a fluid with a viscosity *η* the force law is given by (Clercx and Schram, 1992):

$$m^{*}\frac{dV\left(t\right)}{dt}=-\gamma V\left(t\right)-U^{\prime }\left(X\right)+\eta \left(t\right)-\lambda \overset{t}{\underset{0}{\int }}\frac{d\tau }{\sqrt{t-\tau }}\frac{dV\left(\tau \right)}{d\tau },$$ (32)

where m* = 1 + 0.5*M*_0_/*M* is the ratio of the mass of the Brownian particle *M* to its value shifted by half the virtual mass of sphere of the same size in an incompressible fluid *M*_0_; the ordinary Stokes friction with coefficient *γ* = 6*πηR*/*M* is the first term on the right hand side of Eq. 32; the second term is a mechanical force modeled by the potential function *U*(*X*); the third is the random force generated by the ambient fluid *η*(*t*) = *f*(*t*)/*M*; the final term is the memory associated with the hydrodynamic retardation effects with $\lambda =6R^{2}\sqrt{\pi \rho \eta }/M$ and is today called the *Basset force*. Clercx and Schraom (Clercx and Schram, 1992) solve this equation using Laplace transforms and the time-dependent solution fits experimental data over the entire time domain (Huang et al., 2011).

**FIGURE 6:**
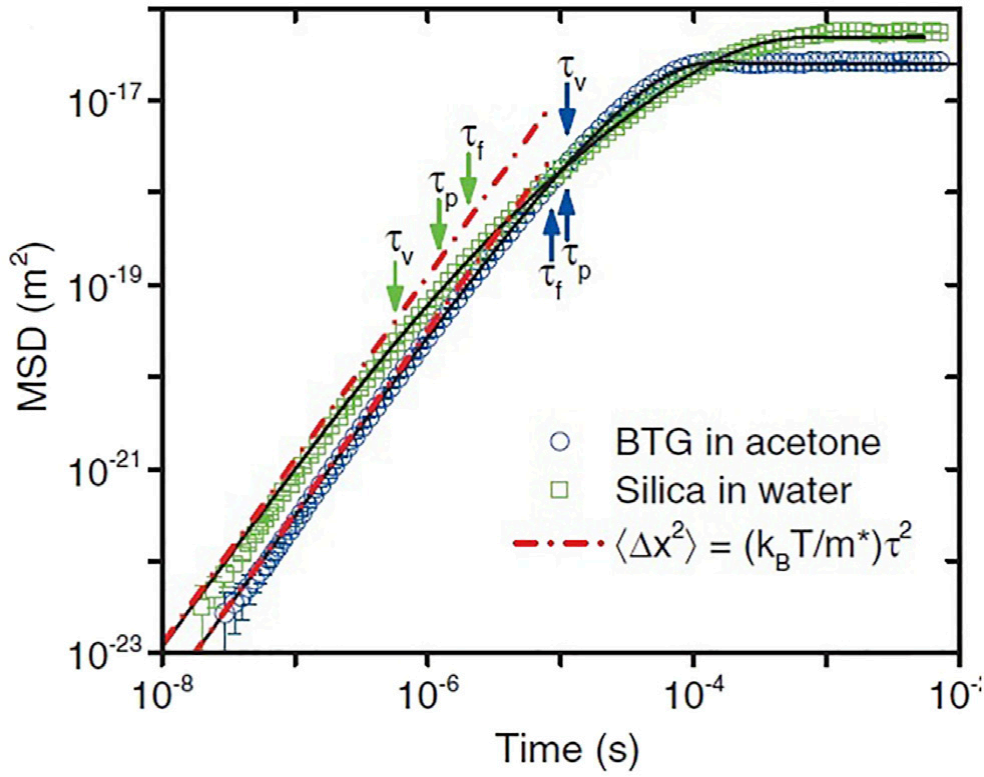
Experimental and theoretical correlation functions from recorded trajectories of two different bead-fluid combinations. Double logarithmic plot of the MSD for an optically trapped barium titanate glass (BTG) bead (3.7 *μm* diameter) in acetone (blue circles; *τ*_*p*_ = 11.0*μs*, *τ*_*f*_ = 8.5*μs*, *τ*_*v*_ = 11.0*μs*), and a silica bead (2.8 *μm* in diameter) in water (green squares; *τ*_*p*_ = 1.2*μs*, *τ*_*f*_ = 2.01*μs*, *τ*_*v*_ = 0.57*μs*). The red dashed lines indicate the MSD of a particle moving at constant velocity. Adapted from (Kheifets et al., 2014) with permission.

Mainardi and Pironi (Mainardi and Pironi, 1996) discuss the solution to Eq.(32) in terms of a FDE:

$$M\frac{dV\left(t\right)}{dt}+\lambda_{0}\partial_{t}^{1/2}\left[V\left(t\right)\right]=-\gamma_{0}V\left(t\right)-U^{\prime }\left(X\right)+\eta \left(t\right),$$ (33)

expressed here in terms of the Caputo fractional derivative $\partial_{t}^{1/2}\left[\cdot \right]$ and the parameters *λ*_0_ and *γ*_0_ are known functions of the fluid viscosity coefficient, the particle masses, and the radius of the Brownian particle, see the excellent paper (Mainardi and Pironi, 1996) for details. The optical trap previously used by the scientists in Raizen’s lab to measure the instantaneous velocity of a harmonically bound particle in air (Li et al., 2010) and a bead in fluid (Huang et al., 2011) was again employed to test the theoretical predictions and interpretations of the Langevin equation modified to include the Basset force. The fit of theory (Clercx and Schram, 1992) to experiment is excellent (Kheifets et al., 2014) as indicated for the fit to the experimental MSD depicted in Figure 6 by the solid black line segments.

We note that fractional Brownian motion and fractional diffusion in general has attracted significant attention recently and now techniques have been developed to identify the fractional diffusion equation from datasets (Znaidi et al., 2020). This is part of the vast literature due to space limitations we can only mention in passing.

#### Inanimate and Living Particles

As emphasized a number of times the FD term in Eq. 33 is the result of the back-reaction onto the Brownian particle by the ambient fluid flowing around it, inducing the retarded viscous force. The solution to this equation is asymptotically dominated by viscous dissipation and the Brownian particle being ‘heavy’ accounts for the success of the usual description of Brownian motion without the inclusion of the FD term. However, when the ambient fluid is not homogeneous, or the Brownian particle is not ‘heavy’, the derivation of the forces acting on the Brownian particle need to be re-examined.

Leptos et al. (2009) conducted experiments on the motion of tracers (Brownian particles) suspended in a living fluid of swimming Eukaryotic micro-organisms of varying concentrations. The interplay between the inanimate Brownian particles and the advection by flows from the swimming micro-organisms results in their displacement having a self-similar PDF with a Gaussian core and exponential tails. Eckhardt and Zammert (2012) re-analyzed these data and obtained an excellent fit to a MLF PDF based on the continuous time random walk (CTRW) model.

A theoretical study of a simplified tracer-swimmer interaction by Zaid et al. (2011) show that the non-Gaussian effect of the tails of the PDF can also arise from a combination of truncated Lévy statistics for the velocity field and the IPL decay of correlations in the ambient fluid. They further show that the dynamics of the PDF leading to the truncated Lévy statistics is given by a fractional diffusion equation, which we discuss subsequently. It is evident that rigorous modeling of Brownian motion in heterogeneous fluids such as microbial suspensions in marine ecologies would potentially benefit from applications of the FDC.

#### Fractional Brownian Motion and the Fractional Calculus

It occurs to me that the above discussion of Brownian motion contained no mention of the fact that one of Mandelbrot’s first formal applications of the fractal concept was to a generalization of this stochastic process. In the paper where Mandelbrot and van Ness (Mandelbrot and van Ness, 1968) introduced the term fractional Brownian motion (*FBM*) they made use of the fractional calculus, but did not think the FC was sufficiently significant to develop further given the context of its utilization and their interpretation of it as a moving average. The fractional operator they used in the definition of *FBM* had been defined by Weyl (1917) in 1917:

$$B_{H}\left(t_{1}\right)-B_{H}\left(t_{2}\right)=\frac{1}{\Gamma \left(\alpha \right)}\left[\underset{-\infty }{\overset{t_{1}}{\int }}\frac{dB\left(\tau \right)}{{\left(t_{1}-\tau \right)}^{1-\alpha }}-\underset{-\infty }{\overset{t_{2}}{\int }}\frac{dB\left(\tau \right)}{{\left(t_{2}-\tau \right)}^{1-\alpha }}\right]$$ (34)

where *dB*(*t*) is a Wiener process, *α* = *H* + 1/2 and as they point out the properties of *FBM* corresponding to the Hurst parameter 0 < *H* < 1/2, 1/2 < *H* < 1, and *H* = 1/2, respectively, differ in many significant ways. This in itself has led to a vast literature some of which are summarized in *Crucial Events* (West and Grigolini, 2021)*.*

### Fractional Differential Equation Models of Cell Growth

In the prequel (West, 2021) we discussed the ubiquity of fractal time series, factal dynamics and fractal geometric structure in physiological phenomena. The analysis gave rise to scaling as a way to directly interconnect the very large with the very small, as well as the very fast with the very slow. The prequel closed with a suggestion that the FC is a systematic way to incorporate spatial inhomogeneity into describing how information is transported across a complex dynamic network. That suggestion was augmented by another involving memory effects in physiologic networks being generic (Goldberger et al., 2002) and the FC was also pointed out by Nasrolahpour (Nasrolahpour, 2017) as being the natural way to incorporate memory effects into the modeling of various complex phenomena including the growth of cancer tumors.

He (Nasrolahpour, 2017) proposed a new model which is a member of a class of simple models that have been extensively used to describe the growth of stem and cancer cells. Following up on his comment about the utility of this mathematical technique in modeling cancer cells I found that over the past decade it had become a cottage industry with hundreds of papers being published each year on the modeling of cancer.

The mathematician Durrett (Durrett, 2013) in a personal perspective on cancer modeling pondered that 80% of the problem is figuring out what the appropriate question is in the biological application and what mathematical tools to use in answering it. This is unlike physics where the applications are typically formulated in such a way that a mathematician may immediately see how s/he might be able to help solve the problem. Or in some cases, like Dirac’s introduction of the delta function in quantum mechanics based solely on his intuition of the physics problem, he saw that the problem required the existence of such an object for its solution. The delta function led to the development of new area of mathematics providing justification of that intuition and provides a useful tool to the broader scientific community once they had caught up with Dirac’s intuition.

With this in mind I have elected to concentrate the following remarks on those aspects of biology and medicine which I believe provide the reasons for FDE being the appropriate mathematics for oncological modeling. We use the master equation for the size distribution of cancer cell colonies *P*(*x*, *t*), defined as the probability that a single cancer cell gives rise to a colony consisting of *x* cells at time *t*. The PDF evolves according to the master equation with nearest neighbor interactions and constant coefficients from (Nasrolahpour, 2017) with a slight change in notation:

$$\frac{dP\left(x,t\right)}{dt}=\left(\begin{array}{ccc} a & -\left(a+b\right)x & b \end{array}\right)\left(\begin{array}{c} P\left(x-1,t\right)\\ P\left(x,t\right)\\ P\left(x+1,t\right) \end{array}\right),$$ (35)

where *b* is the probability per unit time that a cell dies and *a* is the probability per unit time a cell divides into 2 cells. The equation for the growth of the average colony size is obtained by multiplying Eq. 35 by *x* and summing such that after some algebra we obtain the integer rate equation (IRE) for the average size of the colony:

$$\frac{d\left\langle x\left(t\right)\right\rangle }{dt}=\left(a-b\right)\left\langle x\left(t\right)\right\rangle ,$$ (36)

which has the exponential solution for the initial value *P* (*x*, *t* = 0) = *δ*_*x*,1_:

$$\left\langle x\left(t\right)\right\rangle =e^{\left(a-b\right)t}.$$ (37)

Nasrolahpour (2018) states without discussion that his new model of cancer replaces the IRE for the average cancer cell colony size with a FRE. Note that this could be viewed as an adaptation to the cancer problem of the DMM social interaction model introduced in Section 2. We propose doing that here, but instead of an ansatz we apply the *network effect* argument to the interaction of the cell of interest, the one that gives rise to the cancer colony, and transform the IRE into the FRE:

$$\partial_{t}^{\alpha }\left[\left\langle x\left(t\right)\right\rangle \right]=\lambda^{\alpha }\left\langle x\left(t\right)\right\rangle .$$ (38)

Here we have raised the rate *λ* = *a* − *b* to the power of the noninteger derivative *α* in order to retain the same dimensionality on both sides of the equality. Like most differential equations, integer or fractional, we guess the form of the solution and explicitly determine whether or not it solves the equation of interest. The network effect argument applied to a growing population of cells leads to the FDE given by Eq. 14 and the solution Eq. 16 which also solves Eq(38) for *ɛ* = 0.

#### Solving the Linear Fractional Rate Equation

It is time to introduce some of the new mathematics that show how to solve this class of linear FREs. Can we use the Taylor series expansion technique to solve a linear FRE or a more general FDE? The answer is yes, as long as we are sufficiently cautious in doing so. The first caution involves generalizing the Taylor series. We start as with IDEs and introduce a Taylor series for the assumed form of the solution. But with a little thought realize that the Taylor series must be generalized to accommodated the noninteger order of the derivative (West, 2017) in the following way:

$$\left\langle x\left(t,\alpha \right)\right\rangle =\underset{n=0}{\overset{\infty }{\sum }}A_{n}t^{n\alpha },$$ (39)

where we have tagged the proposed solution with the index *α* to match the FRE that is solves. The noninteger derivative $\partial_{t}^{\alpha }\left[\cdot \right]$ in the FRE must satisfy the familiar derivative relation from the ordinary calculus:

$$\partial_{t}^{\alpha }\left[t^{\beta }\right]=\frac{\Gamma \left(\beta +1\right)}{\Gamma \left(\beta +1-\alpha \right)}t^{\beta -\alpha },$$ (40)

when all exponents are integers and $\Gamma \left(\cdot \right)$ is a gamma function. This same relation holds when the exponents are not integers (Podlubny, 1999; West et al., 2003a) in which case substituting the generalized Taylor series into the FRE given by Eq. 38 yields:

$$A_{0}\frac{t^{-\alpha }}{\Gamma \left(1-\alpha \right)}+A_{1}\frac{\Gamma \left(\alpha +1\right)}{\Gamma \left(1\right)}+A_{2}\frac{\Gamma \left(2\alpha +1\right)t^{\alpha }}{\Gamma \left(\alpha +1\right)}+\cdot \cdot \cdot =\lambda^{\alpha }\left\{A_{0}+A_{1}t^{\alpha }+\cdot \cdot \cdot \right\}.$$ (41)

Equating coefficients of equal powers of time from both sides of the equation yields:

$$A_{1}=\frac{\lambda^{\alpha }A_{0}}{\Gamma \left(\alpha +1\right)};\;A_{2}=\frac{\lambda^{\alpha }\Gamma \left(\alpha +1\right)A_{1}}{\Gamma \left(2\alpha +1\right)};\;A_{3}=\frac{\lambda^{\alpha }\Gamma \left(2\alpha +1\right)A_{2}}{\Gamma \left(3\alpha +1\right)};\text{ etc.,}$$

all of which can be generated from the compact form:

$$A_{n}=\frac{\lambda^{n\alpha }}{\Gamma \left(n\alpha +1\right)}A_{0},$$ (42)

and the *n* = 0 term is a tautology. A *A*_0_ term is left over in the process of equating coefficients since the IPL in time given by *t* ^−*α*^ only appears on the LHS of Eq. 41 and must be otherwise accounted for in the analysis. This is the second place where we must be cautious.

Collecting the coefficients from Eq. 42 into the generalized Taylor series after some algebra yields the curious result:

$$\partial_{t}^{\alpha }\left[\left\langle x\left(t,\alpha \right)\right\rangle \right]-\frac{t^{-\alpha }}{\Gamma \left(1-\alpha \right)}\left\langle x\left(0,\alpha \right)\right\rangle =\lambda^{\alpha }\left\langle x\left(t,\alpha \right)\right\rangle ,$$ (43)

and since the *n* = 0 term in the generalized Taylor series is not excluded Eq. 43 is a new FRE. The new FRE explicitly displays the term that was not canceled when the generalized Taylor series was introduced to solve the original FRE and here the unknown coefficient is identified with initial value of the average cancer colony: $A_{0}=\left\langle x\left(0,\alpha \right)\right\rangle$ . With the coefficients inserted into Eq. 39 the generalized Taylor series yields the exact solution to the revised FRE:

$$\left\langle x\left(t,\alpha \right)\right\rangle =\left\langle x\left(0,\alpha \right)\right\rangle \underset{n=0}{\overset{\infty }{\sum }}\frac{{\left(\lambda t\right)}^{n\alpha }}{\Gamma \left(n\alpha +1\right)}.$$ (44)

Remarkably the fractional derivative on the LHS of Eq. 43 has a name, the Riemann-Liouville (RL) fractional derivative (FD), and has a long lineage. To make contact with that long history and for notational clarity we rewrite Eq. 43:

$$D_{t}^{\alpha }\left[\left\langle x\left(t\right)\right\rangle \right]-\frac{t^{-\alpha }}{\Gamma \left(1-\alpha \right)}\left\langle x\left(0\right)\right\rangle =\lambda^{\alpha }\left\langle x\left(t\right)\right\rangle ,$$ (45)

where we have suppressed the *α* − dependence of the solution and added a new symbol $D_{t}^{\alpha }\left[\cdot \right]$ to denote the RL -fractional derivative. The exact solution to the RL FRE is expressed in terms of a series first obtained by the mathematician Mittag–Leffler in the early 20th century and which now bears his name:

$$\left\langle x\left(t\right)\right\rangle =\left\langle x\left(0\right)\right\rangle E_{\alpha }\left({\left[\lambda t\right]}^{\alpha }\right).$$ (46)

The MLF in simplest form is given by the series:

$$E_{\alpha }\left(z\right)=\underset{j=0}{\overset{\infty }{\sum }}\frac{z^{j}}{\Gamma \left(j\alpha +1\right)},$$ (47)

and is here called out explicitly because it appears over and over in both the discussion of applications and in the formal theory of the FDC. Note that the MLF series sums to an exponential for *α* = 1, which accounts for the identical form of the solutions in the linear cooling example. It should also be stressed that like the exponential in the ordinary calculus the MLF appears as the backbone to the solutions of more complicated FDEs, as seen elsewhere (Podlubny, 1999; West, 2016; West, 2017).

Altrock et al. (2015) point out that a branching process is a powerful mathematical tool for the study of cancer population growth. In addition they emphasize that this growth model is based on the assumption that cellular events, such as mutation, replication and death, are independent of one another and is assumed for mathematical simplicity. This independence assumption would be partially removed by taking into account the network effect. The resulting generalization of Eq. 46 would be the case where, at any time, each cell is fully described by cell-intrinsic probability rates of proliferation, mutation, and death, as well as the parameter of the FRE noninteger order. The latter parameter *α* provides a measure of the level of internal dependency of the intrinsic dynamic processes.

### Tumor Nonlinear Dynamics

In the previous subsection we took advantage of the linear master equation to show how probabilistic arguments can be generalized using the FC for a linear dynamic system. In the present section we take a different tack and instead briefly review a number of growth models that have been borrowed from the social sciences and adapted for the modeling of tumor growth. Each of these borrowed and adapted models is nonlinear and that provides a new degree of difficulty in solving the resulting equations. Recall that the FRE obtained from the network effect took such nonlinearities into account without explicitly linearizing the full dynamics of a network.

The most famous of the nonlinear growth equations was introduced into social science by Verhulst in 1838 in order to provide a rationale for a way to limit the world’s population and thereby alay the fears resulting from the dismal forecasts of Maltus, who predicted unflagging exponential population growth that would all too soon quench the world’s linearly growing food supply, resulting in world-wide famine. The nonlinear equation of Verhulst has become known as the logistic growth model and as pointed out by Varalta et al. (2014) has been used to successfully describe the growth of populations in both the laboratory and in natural habitats, limiting the growth by influencing factors of competition, mortality and fertility. As more complex effects enter into the modeling, such as interactions within food webs, a number of investigators have generalized the logistic equation using the FC to help slow the convergence to the population’s carrying capacity.

Much of the previous work in this regard has been on the numerical simulation of fractional nonlinear growth models (FNGMs) and a number of these numerical methods were used to test analytic results, see e.g., the 51 papers devoted to the *Future Directions in FC Research and Applications* (Meerschaert et al., 2017) as an exemplar of the rigorous mathematics being done in this area.

#### Fractional Logistic Equation

Varalta et al. (2014) are investigators whose work bridges the gap between the complexity of medicine and the mathematics on which medical models can be based. The Malthusian model of exponential growth flies in the face of observation, whether it is the growth of a population of humans or of cells. What all growth processes have in common is that the population must be continually supplied with nutrients, the individual members must be born and they eventually die. This is the process that Malthus modeled and is incorporated into the master equation Eq. 35 with a modest generalization in the form of the distribution of possible futures, but with the pessimism of Malthus being the final average outcome. Verhulst introduced the idea that a society has a finite carrying capacity, such that the rate of growth is dependent on the population and this rate goes to zero as the carrying capacity of the population is approached:

$$\frac{dX\left(t\right)}{dt}=\lambda \left[1-X\left(t\right)\right]X\left(t\right),$$ (48)

where if *N*(*t*) is the instantaneous population and *N*_*T*_ is the carrying capacity (the maximum population the society can maintain) we have *X*(*t*) ≡ *N*(*t*)/*N*_*T*_. The Verhulst or logistic equation is popular because it: 1) provides a reasonable explanation for why the exponential growth is suppressed; 2) the growth is eventually sub-exponential and saturates at a finite value; 3) the logistic equation has an analytic solution.

The analytic solution to the logistic equation is obtained by making the substitution of variables *Y*(*t*) = 1/*X*(*t*) to obtain the linear growth equation:

$$\frac{dY\left(t\right)}{dt}=\lambda \left[1-Y\left(t\right)\right],$$ (49)

which is solved in terms of *e* ^*λt*^ and the initial population *X* (0). Inverting the substitution variable in the solution to this linear equation yields the sigmoidal solution to the logistic equation:

$$X\left(t\right)=\frac{X\left(0\right)}{e^{-\lambda t}+\left(1-e^{-\lambda t}\right)X\left(0\right)}\underset{t\to \infty }{\to }1,$$ (50)

which asymptotically approaches the carrying capacity, which is 1 in these units.

There are a number of ways to introduce the fractional calculus into the logistic model of growth. One way is through the introduction of the Carleman embedding technique, which follows from the theorem that any finite order nonlinear equation of motion can be replaced by an infinite order set of linear equations (West, 2015). Another is a spectral technique to solve a nonlinear FDE (Turalska and West, 2017). Both these approaches start from the fractional logistic equation:

$$\partial_{t}^{\alpha }\left[X\left(t\right)\right]=\lambda^{\alpha }\left[1-X\left(t\right)\right]X\left(t\right),$$ (51)

where one might have used the subordination method introduced in Section 2.1 to replace the integer order time derivative with the Caputo time derivative. We now know that the fractional derivative in time incorporates memory into the population dynamics. Both the Carleman embedding and spectral techniques yields the same solution to the fractional logistic equation (Turalska and West, 2017):

$$X\left(t\right)=\underset{n=0}{\overset{\infty }{\sum }}{\left(\frac{X\left(0\right)-1}{X\left(0\right)}\right)}^{n}E_{\alpha }\left(-n\lambda^{\alpha }t^{\alpha }\right),$$ (52)

which is an expansion over a set of eigen functions given by the MLFs, and the coefficients are in terms of powers of the initial value. Note that asymptotically all the MLFs go to zero except the *n* = 0 term which yields the carrying capacity of the network. The choice *α* = 1 reduces the MLF to the exponential *e* ^*nλt*^ and the sum over eigen functions in Eq. 52 reduces the solution to the ordinary logistic equation given by Eq. 50.

**FIGURE 7:**
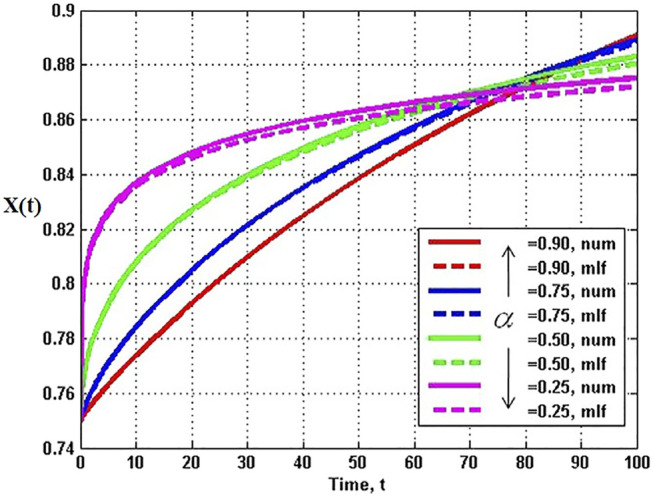
Analytic solutions to the fractional logictic equation (solid) for a number of fractional derivatives are compared to the numerical solutions (dashed) for the values of the fractiona-order *α* indicated with *X* (0) = 0.75, *λ* ^*α*^ = 0.1, and time step 0.001. From (West, 2015) with permission.

The analytic series solution to the fractional logistic equation given by Eq. 52 is compared with the numerical integration of the FDE in Figure 7 each for the same time step and initial condition. It is evident by inspection that the closer the fractional order is to unity the closer the correspondence between the analytic and numerical results. There are some technical issues with this solution which are addressed in (Turalska and West, 2017), but their discussion would take us too far into the mathematical weeds to be of value here.

A very different way of introducing the influence of the FC on the logistic growth is made by (Varalta et al., 2014). They cleverly introduce the fractional derivative into the linear growth of the transformed variable *Y*(*t*), which was introduced in Eq. 49 rather than in the nonlinear equation given by Eq. 51. This choice for the insertion of the fractional derivative essentially introduces the network effect into the time rate of change of the population-dependent growth rate:

$$\partial_{t}^{\alpha }\left[Y\left(t\right)\right]=\lambda^{\alpha }\left[1-Y\left(t\right)\right],$$ (53)

rather than into the population dynamics directly. Taking the Laplace transform of the linear equation, after some algebra the Laplace equation can be inverted to yield the solution to the initial value problem:

$$X\left(t\right)=\frac{X\left(0\right)}{E_{\alpha }\left(-\lambda^{\alpha }t^{\alpha }\right)+X\left(0\right)\left[1-E_{\alpha }\left(-\lambda^{\alpha }t^{\alpha }\right)\right]}.$$ (54)

However, these solutions are equal when *α* = 1 and MLF becomes an exponential, just as did Eq. 52 even though these two solutions appear to be very different from one anothe for *α* < 1.

These authors (Varalta et al., 2014) point out that the use of such sophisticated mathematical techniques in the modeling of medical pathologies is of recent vintage, particularly in the study of cancer tumors. They consider this to be one of the reasons that the methods are still finding difficulty in modeling the growth of tumors satisfactorily:

In the case of tumor dynamics saturation of various types of tumors is not well modeled by the exponential model. For this reason, this model applies only to avascular tumors, i.e., when angiogenesis has not occurred…Indeed, tumor cells compete for oxygen and vital resources that is the reason why the logistic model fits well in several cases…

We can here answer the question as to whether the fractional forms of the logistic equation and that of the linear form in the transformed variable have the same solution. Taking the solution given by Eq(54) and expanding it is an infinite series yields:

$$X\left(t\right)=\underset{n=0}{\overset{\infty }{\sum }}{\left(\frac{X\left(0\right)-1}{X\left(0\right)}\right)}^{n}{\left[E_{\alpha }\left(-\lambda^{\alpha }t^{\alpha }\right)\right]}^{n},$$ (55)

and the inequality:

$$E_{\alpha }\left(-n\lambda^{\alpha }t^{\alpha }\right)\neq {\left[E_{\alpha }\left(-\lambda^{\alpha }t^{\alpha }\right)\right]}^{n},$$ (56)

establishes proof that the solutions given by Eqs. 52 and 54 are not the same. The relation between the MLFs becomes an equality in the singular case *α* = 1 and the MLF becomes an exponential. However, this does not tell us which of the two models better describes the growth of tumors. In fact since there is no universal law to describe tumor growth these two contenders remain in competition with a host of others, all of which await the detailed fit to extended datasets.

### Mathematical Oncology and Fits to Data

Oncology is the branch of medicine that deals with the treatment, diagnosis, and prevention of cancer and the models of tumor growth are within the ever broadening domain of MO. As pointed out by Valentim et al. (2020) all solid cancers originate with the growth of a primary tumor, and the majority of the growth patterns follow a sigmoidal shape determined by the population’s growth rate and carrying capacity. They go on to argue that the IDE models for tumor growth possesses certain deductive-reductionistic characteristics that are maintained when such models are generalized to fractional form, for example, the inclusion of memory and heterogeneity effects in fractional MO (FMO).

Valentim et al. (2020) study the deviation in tumor growth from a simple exponential for the analytic solutions for four distinct nonlinear growth models in the IDE as well as the solutions to their fractional generalizations. The FDE and IDE $\left(\alpha =1\right)$ models considered have the generic form:

$$\partial_{t}^{\alpha }\left[u\left(t\right)\right]=af\left(u\right)-bg\left(u\right),$$ (57)

here *V*(*t*) is the size of the tumor volume at time *t*, the two functions *f* and *g* determine the functional form of the growth: fractional exponential *u* = *V*, *f* = *V*, *g* = 0; fractional logistic *u* = 1/*V*, *f* = *au*, *g* = 1; fractional Gompertz *u* = *lnV*, *f* = 0, *g* = *u*; fractional Bertalanffy *u* = *V* ^*p*^, *f* = *p*, *g* = *pu* where *p* is a rational fraction. The solutions to the exponential and logistic forms have been presented in previous sections and the remaining models are solved using the methods discussed and may be expressed in terms of MLFs (Valentim et al., 2020).

**FIGURE 8:**
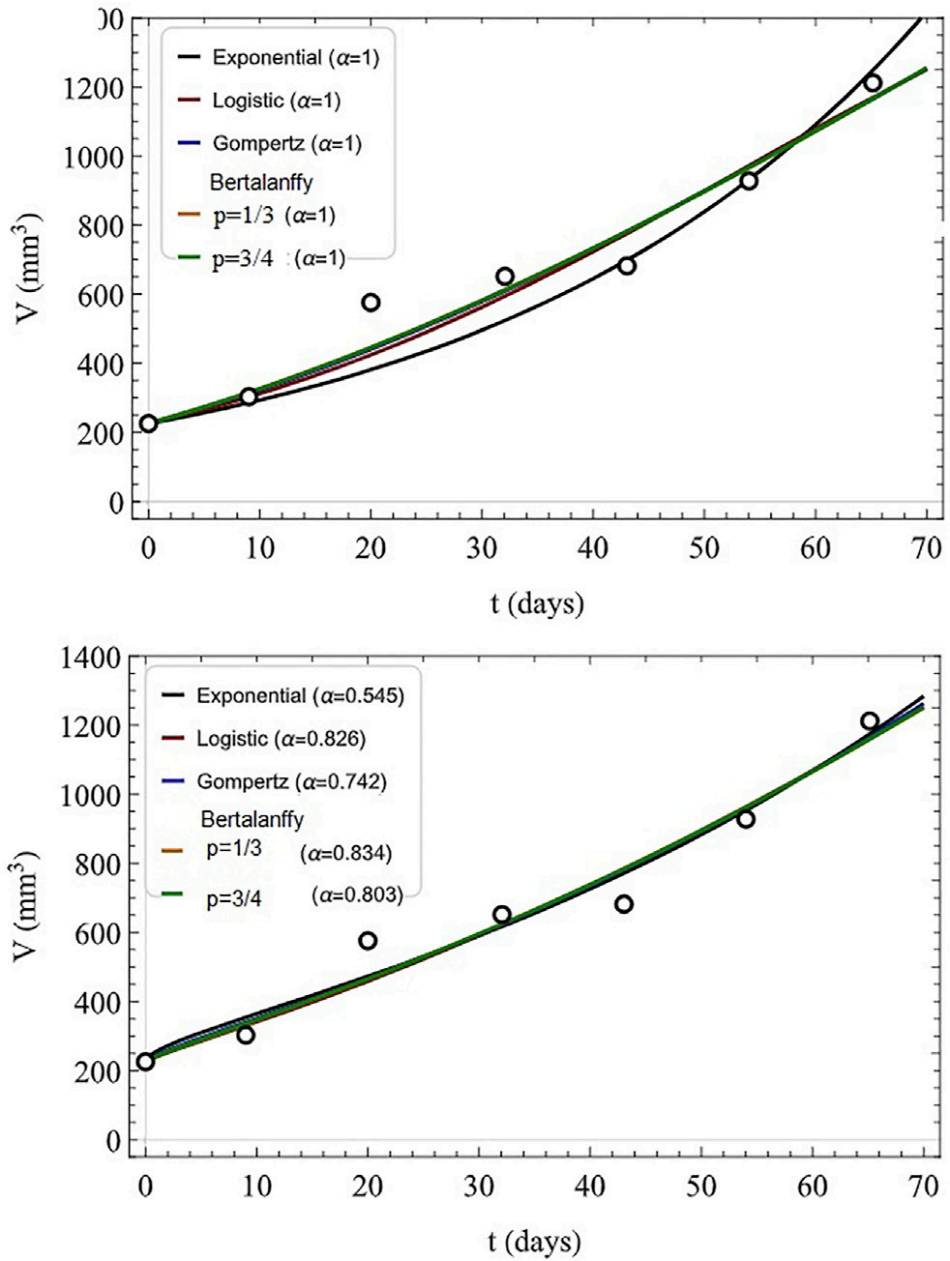
Fitted tumor growth: clinical data (circles), numerical fits to data (first seven data points). The models used are indicated for IDE (top, *α* = 1) and FDE with indicated values of *α* (bottom). Adapted from (Valentim et al., 2020) with permission.

The algebraic form of the various solutions are not as important as their flexibility in fitting tumor growth datasets. Such fits are indicated in Figure 8 where the first seven tumor volume data points are indicated along with fits to the IDE models (top) and their FDE generalizations (bottom).

**FIGURE 9:**
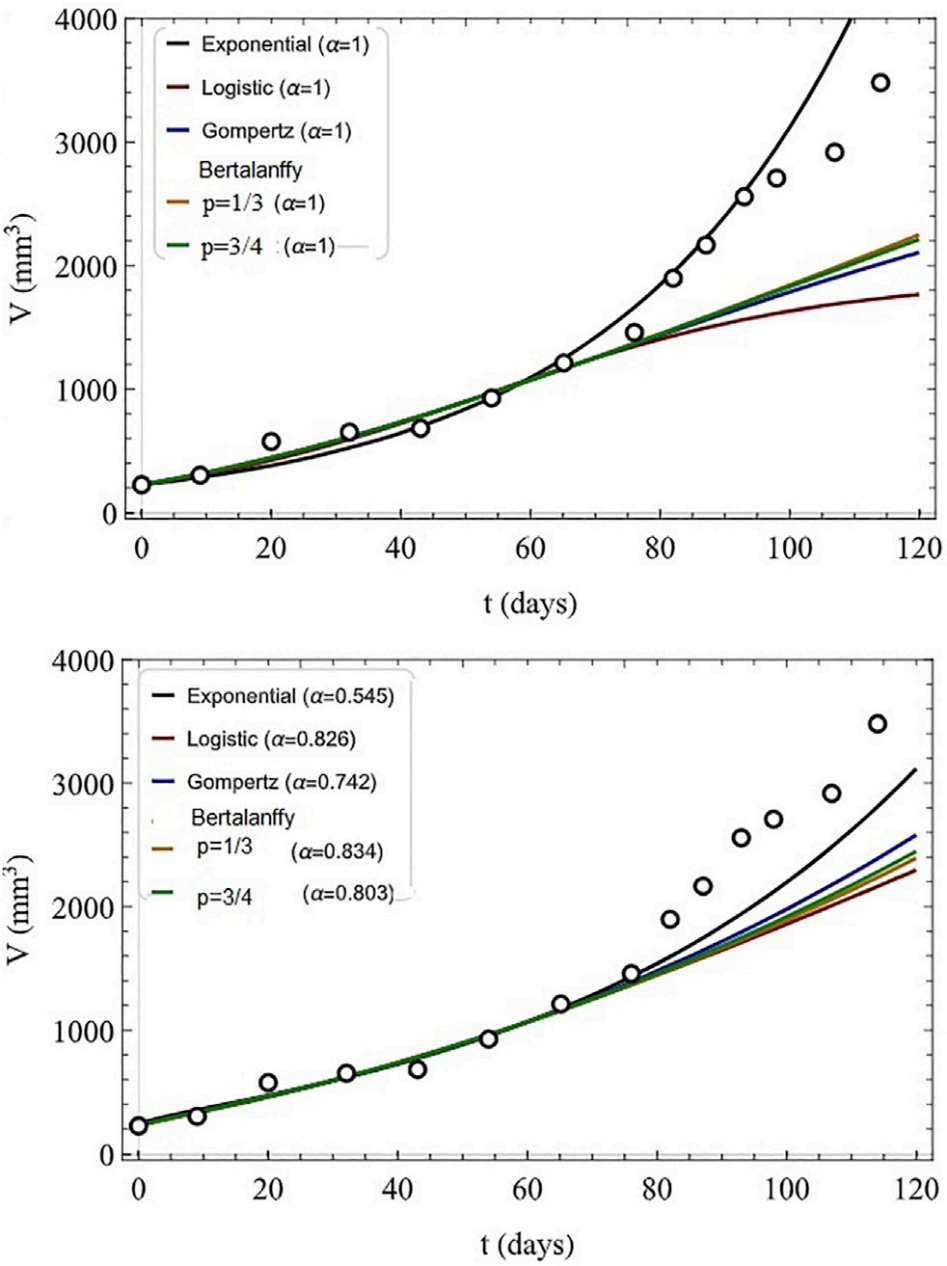
Predicted tumor growth: clinical data (circles). The models used are indicated for IDE (top, *α* = 1) and FDE with indicated values of *α* (bottom) and the last seven data points are predicted using the model parameters obtained in Figure 8. Adapted from (Valentim et al., 2020) with permission.

Using the parameters fit of the first seven data points to the model parameters the prediction of the next seven data points are indicated in Figure 9 with the IDE models (top) and their FDE generalizations (bottom). Valentim et al. (2020) discuss the relative merits of the modeling of tumor growth using integer versus fractional time derivatives:

If one also considers fractional models instead of only their classical versions, the indicator for how close the best model replicates experimental data would rise from 67.5 to 88.8% - a very significant improvement. This reveals a major convenience of using fractional models as they keep a higher degree of information regarding the fitted time series, decreasing the chance of misfitting while still maintaining a relatively simple and reductionist form. Such advantage is mainly attributed to the memory effect, a characteristic inherently linked to the definition of fractional operators allowing models to consider not only elements at the evaluation instant but also those occurring before. This feature naturally favors fractional models to describe biological phenomena.

This improvement in the mean-square error supports the interpretation that the FDE models include more information of the cancer time series being fitted than do the IDE models.

In spite of the positive observations made regarding the fractional models it is evident from the comparison with real data that neither the IDE nor FDE models provide an accurate prediction of the asymptotic size of the tumor. On the other hand, even slightly better predictions may improve clinical assessments so the choice of FDE model should be made very carefully (Valentim et al., 2020).

Our intent here is not to argue for the superiority of one numerical fitting technique over another. Rather it is to provide insight provided by a new mechanism available to a FDE model that is not reachable using IDE models. The fitting of the fractional derivative order to the first seven data points provides a mechanism not available to IC fitting procedures. This fitting of the order of the fractional derivative to the early data means that any early change in the internal dynamics can be captured and influences the later system behavior.

The closest analog to this situation in the IC modeling of complex systems is the telegrapher’s equation (TE). The diffusion process is generalized in two important respects for telegraphic processes: 1) the TE allows for a finite velocity of information propagation which is infinite in the diffusion equation and 2) the TE at short times describes nearly deterministic wave propagation, whereas at long times the TE supports diffusive behavior.

In the one-dimensional case the MSD for the solution to the time-fractional TE at early times describes wave motion with damping and at late times diffusion with a finite velocity (Masoliver, 2021):

$$\left\langle X{\left(t\right)}^{2}\right\rangle \left\{\begin{array}{c} ∼t^{2\alpha },\;t\to 0\\ ∼t^{\alpha },\;t\to \infty . \end{array}\right.$$

For the IC TE we have *α* = 1 and obtain the familiar IC results. When generalized to the fractional order TE with *α* < 1 the solution is a bi-fractal with the fractal dimension halving from one asymptotic time regime (*t* → 0) to the other (*t* → *∞*). We refer the interested reader to the excellent review by Masoliver (Masoliver, 2021) for details and we shall have more to say on the notion of multifractal behavior and its relation to FC subsequently.

#### Variable Fractional Order

The fitting of the fractional growth models to the tumor dataset has so far not exploited the full flexibility of the FC. We have replaced the IDE growth models, both linear and nonlinear, with their FDE generalizations. In making these replacements we have used two distinct arguments. One based on the network effect and the other on the time subordination method. We now examine an additional generalization of both these techniques and consider what might make the fractional order of the time derivative itself a function of time, that is, what property of tumor growth would entail:

$$\partial_{t}^{\alpha }\left[\cdot \right]\to \partial_{t}^{\alpha \left(t\right)}\left[\cdot \right],$$ (58)

where $\alpha \left(t\right)$ is a time-dependent fractional order derivative and is called a time profile in (Valentim et al., 2021). Such a time-dependency could incorporate into the growth process the changes in physical characteristics and biomechanical modifications that tumors undergo while advancing toward their malignant final state.

When a mechanical force is applied to a solid body that body changes shape in response to the applied force; these deforming forces are collectively called *stresses* (*σ*) and the deformation the body undergoes under stress is called *strain* (*ɛ*). An object that undergoes deformation can do one of two things after the stress is released. An elastic material object returns to its original shape immediately, whereas a plastic material object will retain its deformation for some length of time including permanently. The branch of physics dealing with study of the dynamics of plastic materials goes by a number of different names including rheology, viscoelasticity and hereditary solid mechanics, all of which address the solution of dynamic stress-strain relations. It is in this context that the time-dependent fractional derivative expressed in Eq. 60 has been most fully motivated for application in MO (Di Paola et al., 2020; Valentim et al., 2021).

Di Paola et al. (2020) note that the response of a linear viscoelastic material to a generic imposed strain or stress history is obtained by applying Boltzmann’s linear superposition principle:

$$\begin{array}{ccc} \sigma \left(t\right) & = & \overset{t}{\underset{0}{\int }}\Phi \left(t-\tau \right)d\varepsilon \left(\tau \right), \end{array}$$ (59)

$$\begin{array}{ccc} \varepsilon \left(t\right) & = & \overset{t}{\underset{0}{\int }}J\left(t-\tau \right)d\sigma \left(\tau \right), \end{array}$$ (60)

where $\Phi \left(t\right)$ is the relaxation function from a constant strain $\varepsilon \left(t\right)=\varepsilon_{0}\Theta \left(t\right)$ , and *J*(*t*) is the creep function for a constant stress $\sigma \left(t\right)=\sigma_{0}\Theta \left(t\right)$ and $\Theta \left(t\right)$ = 1 for *t* ≥ 0, and = 0, for *t* < 0. Under the linearity assumption these expressions are readily extended to complex FDE form with a constant power law index, see e.g., West et al. (2003a). In the linear regime the creep law is:

$$J\left(t\right)=\frac{t^{\alpha }}{E_{\alpha }\Gamma \left(1+\alpha \right)},$$ (61)

and the relaxation function is:

$$\Phi \left(t\right)=\frac{E_{\alpha }t^{-\alpha }}{\Gamma \left(1-\alpha \right)},$$ (62)

and 0 < *α* < 1 and the standard generalizations to the FC for the stress-strain relations can be made. Note that this is the physical basis for the failure of linear IDEs to describe what appeared to be simple phenomena until the environment was more completely analyzed and found to introduce either memory or spatial heterogeneity into the dynamics.

However even the more familiar arguments breakdown when the dynamics become nonlinear, because in that situation the Boltzmann superposition principle no longer applies. However, if *α* and $E_{\alpha }$ are constant and the nonlinearity involves only the level of stress, the Boltzmann superposition principle holds in a space different from the traditional $\left(\varepsilon ,\sigma \right)$ -space and the response properties may be represented by the FC operations of constant order. As stated by Di Paola et al. (2020):

However, for the purpose of handling systems where $E_{\alpha }$ and *α* may change during the time interval of interest new and pertinent fractional calculus tools should be considered rather than variable-order fractional-operators, as obtained from classical fractional operators upon replacing the constant order with a variable one; indeed, these operators implicitly rely on the assumption that the Boltzmann linear superposition holds true in the classical form, which may not be a rigorous assumption in the presence of the nonlinearity associated with changing values of *α*.

The approach developed by Di Paolo et al. is not presented here due to space constraints, but does warrant a number of additional comments. As they point out their proposed approach is an effective way to build the stress (strain) response of a nonlinear viscoelastic material body having time-dependent fractional order operators to a general imposed strain (stress) history. Throughout the observation time interval it is assumed that the evolution of the parameters *α* and $E_{\alpha }$ are known at each instant of interest.

**FIGURE 10:**
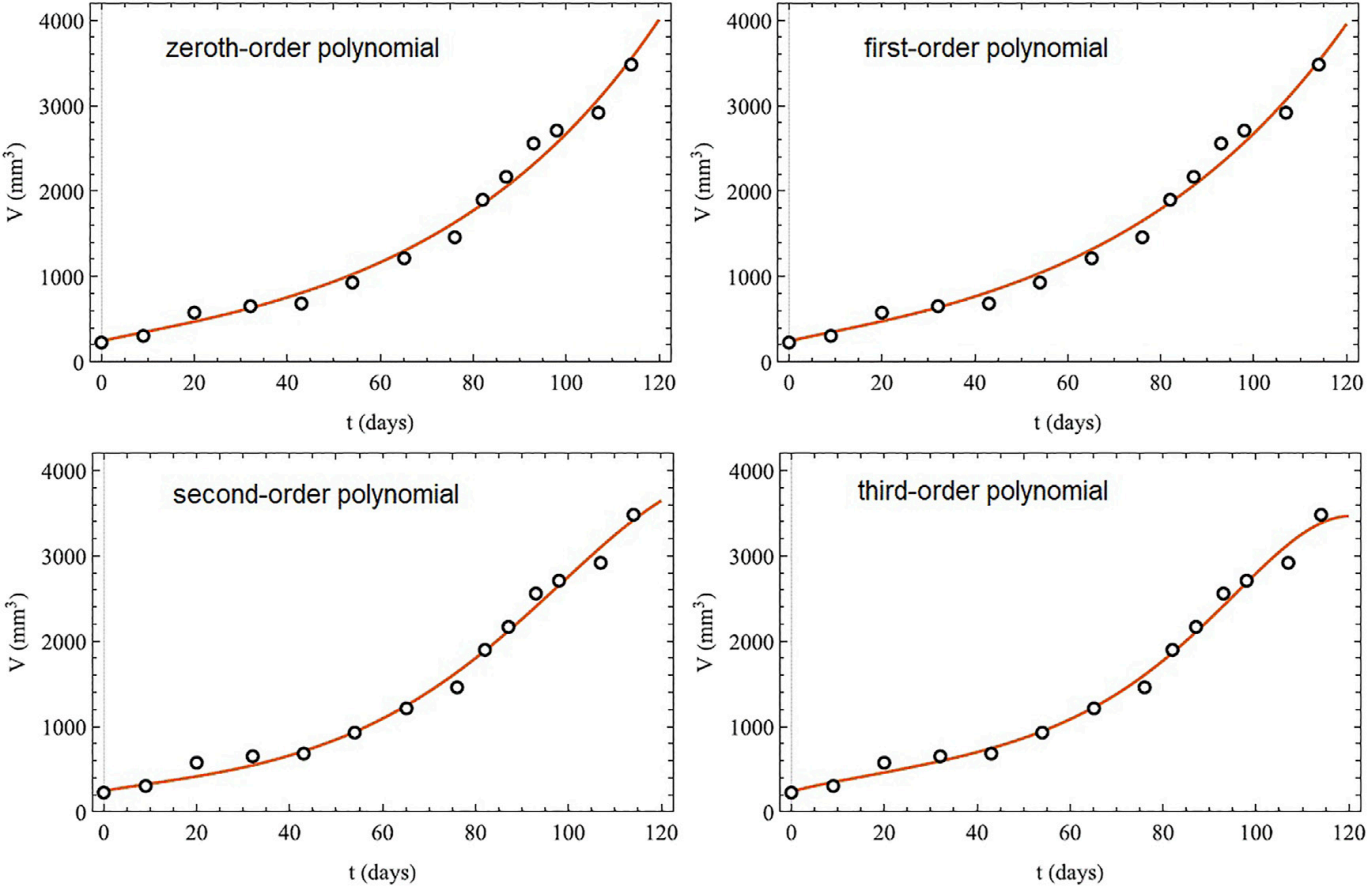
Tumor growth: comparison between clinical data (open circles) and the best-fit variable-order models (solid line segment) given by the MLF solution Eq. 64 with *λ* = 0.090 8. The time-varying order is given by the Taylor series with N terms Eq. 63. Adapted from (Valentim et al., 2021) with permission.

Here we demonstrate the utility of a time-varying order *α*(*t*) using the previously fitted tumor growth dataset. This is done even though such a mathematical description of specific time-dependent tumor features are not known *a priori*. Valentim et al. (2021) use an exploratory approach and capture the 14 data point history of the tumor in the value of the order of the time derivative with a Taylor series:

$$\alpha \left(t\right)=\underset{n=0}{\overset{N}{\sum }}\alpha_{n}t^{n},$$ (63)

where the *n* = 0 coefficient corresponds to the fixed-order FC model. The FRE for tumor growth is given by Eq. 38 with time-varying order and has the MLF solution for the growth of the tumor volume *V*(*t*):

$$V\left(t\right)=V_{0}E_{\alpha \left(t\right)}\left(\lambda t^{\alpha \left(t\right)}\right),$$ (64)

where the MLF has the form defined by Eq. 47. The fitting of the MLF solution with time-varying order to the 14 data points is given in Figure 10 for four consecutive orders of the polynomial in the Taylor series. The variable-order models fit the dataset better than either the solution to the IRE or to the FRE with errors to the fit that decrease with the order of the polynomial N in Eq. 63.

Note the two very different strategies for fitting the same dataset in Figures 9, 10. The former uses a nonlinear FRE with a fixed order whose numerical value varies with the model nonlinearity to fit the early time well but does not do well in predicting the tumor volume at late times. The latter multistep experimental model tunes the profile of the time varying order to a third-order polynomial that appears to capture the nuances of the pattern of growth of the tumor volume extremely well. Valentim et al. (2021) emphasize that these latter results indicate the superiority of the proposed strategy for describing experimental data and provides a new perspective for modeling tumor growth. They also explain the difference between a time varying rate of growth *λ*(*t*) and the variable-order *α*(*t*). While it is true that the time variable order does in fact ultimately dictate the time changes in the growth rate the biomedical interpretation of *α*(*t*) goes a great deal deeper. A possible source of the time-variability is the fractional stress-strain relations just discussed to take into account the tumor evolution, but in addition how that influence may change during the separate growth stages.

Valentim et al. (2021) make a strong case for interpreting $\alpha \left(t\right)$ as a memory index, which may potentially translate the time variation to the activation and/or development of identifiable hallmarks of tumor evolution:

When *α* ≈ 1 tumors follow an exponential increase “programmed” in their original cells (activating hallmarks related to the evasion of growth suppressors and sustainability of proliferative signaling). On the other hand, when *α* is lower tumors evolve at a slower growth rate, potentially due to challenges from the microenvironment (e.g., shortage of nutrients, extracellular matrix resistance). In this case, they “forget” (or inactivate) part of their original programming, developing traits suitable to their current evolution stage (hallmarks related to angiogenesis and invasion).

### Distributed Fractional Order Derivative and Multifractality

Another of the avenues we have not explored is signal processing and here again Mandelbrot was the first to recognize that signals that are singular at almost every point are encountered everywhere including in physiological datasets. One historical strategy for interpreting a signal in communication theory is to construct the reciprocal integral relations of Fourier and although the method is mathematically unassailable the interpretation of the various derived quantities have been called into question. The basis for these questions is the mutually exclusive treatment of time and frequency in the specification of the signal, that is, the time series is assumed to have infinite length and each frequency is defined for an infinite monochromatic wave train. In the real world, particularly in medicine, all time series are of finite duration and frequencies change over time. The recognition of this limitation of the (time, frequency) -representation of Fourier signals led to the development of the *wavelet transform* method for representing one-dimensional signals as a function of time and frequency (Mallat, 1999).

Here let us define the wavelet transform *T*_*g*_ (*a*, *b*) of a time series *X*(*t*) with respect to a wavelet *g*, which is broadly interpreted as being equivalent to a mathematical microscope whose magnification is 1/*a*, whose position in the time series is *b*, and whose optics are given by the choice of the specific wavelet function *g* (West, 1990). I bring this up here because one can relate this formalism to the FC by applying a wavelet transform to a fractal function, say a function describing the growth of a tumor, as we sketch below.

A fractal function is self-affine and can be generalized by examining the local scaling properties of the function at small scales. Consider an arbitrary point *t*_0_ in the time series *X*(*t*):

$$X\left(t_{0},t\right)=X\left(t+t_{0}\right)-X\left(t\right),$$ (65)

and the function remains the same up to a scale factor at different length scales. In this case self-affinity at the point *t*_0_ < *t* means that by scaling the local variable *t* with a parameter *λ* > 0 yields:

$$X\left(t_{0},\lambda t\right)=\lambda^{\alpha \left(t_{0}\right)}X\left(t_{0},t\right).$$ (66)

The parameter *α* is the local scaling exponent at *t*_0_ and can be shown to be the fractal dimension of the process being measured *X*(*t*). The fact that *α* is a function of *t*_0_ means that the time series *X*(*t*) is multifractal; when the scaling index is independent of *t*_0_ then *X*(*t*) is a homogeneous fractal. Equation 66 was established using the wavelet transform to construct the scaling relation (West, 1990):

$$T_{g}\left(\lambda a,\lambda b+t_{0}\right)X\left(t\right)=\lambda^{\alpha \left(t_{0}\right)+1/2}T_{g}\left(a,b+t_{0}\right)X\left(t\right),$$ (67)

thereby establishing the underlying process to be multifractal.

Halsey et al. (1986) posit that if a dynamic system is partitioned into pieces of size *l* then the number of times the exponent takes on the value *α* is:

$$N\left(l\right)∼l^{-f\left(\alpha \right)},$$ (68)

where *f*(*α*) is the continuous spectrum of singularities of strength *α*. They go on to show that the generalized fractal dimension *D*_*q*_ can be computed directly from the singularity spectrum:

$$q=\frac{df\left(\alpha \right)}{d\alpha }\;\to D_{q}=\frac{1}{q-1}\left[q\alpha -f\left(\alpha \right)\right],$$ (69)

and consequently the scaling properties of the multifractal is determined by:

$$\alpha =\frac{d}{d\alpha }\left[\left(q-1\right)D_{q}\right].$$ (71)

Measurements of the *D*_*q*_’s and the spectrum of singularities provide global and statistical information of the scaling properties of fractal measures. This information is similar to the power spectral density obtained from the Fourier transform of a time series that quantifies the relative contributions of the underlying frequencies. The function $f\left(\alpha \right)$ quantifies the relative contribution of the underlying singularities. Just as the Fourier transform does not keep track in time of the frequencies contributing to a power spectral density, neither does $f\left(\alpha \right)$ denote the locations of the singularities. Thus, a multifractal spectrum can only give an indication of the span of dimensions being accessed by the dynamic process and not the order in time at which they occur.

A vast literature has become available on multifractals and their processing techniques over the last quarter century, and there is every indication that the complex dynamics evident in tumor growth from early insights (Baish and Jain, 1998; Nonnenmacher et al., 1993; Losa et al., 1998; ibid, 2002; ibid, 2005; Meakin, 1998) to the development of analytic methods based on multifractal analysis to characterize the emergent properties of complex biological patterns (Balaban et al., 2018) will be facilitated by multifractal data processing techniques (Ivanov et al., 1999; Ivanov et al., 2009).

## OM and Diffusion

No discussion of OM would be complete without at least a brief review of the phenomenon of diffusion in a reaction-diffusion type of modeling of cancer growth, even without an extension beyond IDEs to include the fractional derivatives in space as well as in time. An excellent review of diffusion starting from the simple Brownian motion of tracer particles but focusing on the deviations from the laws of Brownian motion is given by Metzler et al. (2014), who provide an overview of different popular anomalous diffusion models paying special attention to their loss of ergodic properties. They highlight several of these models concentrating on the long-time averaged mean squared displacement, showing that the data obtained from time averages are different from ensemble averages. Thus, the workhorse of statistical physics, the ergodic hypothesis, breaks down.

The oncological application of anomalous diffusion is made by Debbouche et al. (2021), who remark that the first mathematical tumor growth models were integer partial differential equation (IPDE) models taking into account tumor, normal and dead cells, nutrition, various inhibitory substances and immune system response. Cells of a healthy organism are mortal with apoptosis being the end of the life cycle, whereas the lack of apoptosis is a main feature of tumor cells.

Here we take a different route and offer various mechanisms that modulate the diffusion process as well as compliment the growth laws already discussed. In this section we are concerned with characterizing the growth of a single species, both in isolation in the presence of other species. The growth of a species in isolation is modeled by means of a growth function which is intended to represent the influence of the fluctuations in the environment on the population. The influences being modeled will, of course, vary from species to species and will often be left quite general in the discussion so as not to unnecessarily limit the applicability of the growth function being used. The total rate of growth of a species is only partly given by such a function. As the population grows in a region of space, it may also migrate into the surrounding territory; also, members in other regions of space may migrate into the given region. This migration may be motivated by the avoidance of competition for common food sources with other species, the depletion of local food stuffs and/or becoming the food source of another species.

Our discussion centers on the three components of the multi-species network: 1) the growth in time of a single species in isolation, 2) the diffusion of that species in space, and 3) the modification of the growth and redistribution (diffusion) of a given species due to its interaction with other species.

### Diffusion, Growth and Interactions

The non-homogeneous diffusion equation for the density of cells is given by:

$$\frac{\partial \rho \left(x,t\right)}{\partial t}=D\frac{\partial^{2}\rho \left(x,t\right)}{\partial x^{2}}+f\left(x,t\right),$$ (71)

where $\rho \left(x,t\right)$ is the concentration of cells at spatial location *x* and time *t* and *D* denotes the diffusion coefficient throughout this chapter*.* When we consider a specific type of cell the concentration will carry an index to indicate the cell type, and the initial state will be denoted by $\rho \left(x,t=0\right)=\rho_{0}\left(x\right)$ . The non-homogeneous term is a growth function for the concentration that we write as:

$$\begin{array}{ccc} f\left(x,t\right) & = & \rho \left(x,t\right)G\left(\rho \right), \end{array}$$ (72)

$$\begin{array}{ccc} G\left(\rho \right) & \equiv & \kappa \frac{1-\rho {\left(x,t\right)}^{\mu }}{\mu }, \end{array}$$ (73)

and logistic growth is identified with *μ* = 1 and the Gompertz growth with the limit *μ* → 0, although the latter equation was developed to model the mortality of the elderly rather than population growth.

The Verhulst and Gompertz models, as well as those with more general forms of $G\left(\rho \right)$ were constructed to predict the growth of a single species at a rate *κ* in a stable environment with limited resources. A growing population can circumvent the saturation inherent in such growth laws (*D* = 0) by redistributing (diffusing *D* ≠ 0) into nearby unoccupied territory as the resources become depleted at their existing locations. The diffusion does not stop the saturation but it does retard it. These complementary mechanisms are subsequently discussed in the oncology context.

We can express the self-limiting growth equation by neglecting diffusion and setting *D* = 0 in Eq. 71:

$$\frac{dN\left(t\right)}{dt}=NG\left(\frac{N}{N_{T}}\right),$$ (74)

and *N*_*T*_ is the carrying capacity of the growth model, i.e., *ρ* = *N*/*N*_*T*_. The first restriction we relax here in the discussion of Eq. 74 is that of the stability of the environment. For example, in the case of human populations, such things as a vacillating economy or war could have extremely large effects on the population; whereas for lower-level biological species, violent weather changes or short-term food shortages could be major influences in the population’s growth. Finally, a tumor’s environment may disrupt the growth by invading with blood vessels as well as other things. Since it is their unpredictability which all these impacts have in common, the most elementary way to model their effects and still maintain a degree of generality is to assume them to be random. We model these external influences by adding a random forcing term *F*(*t*) to the IRE, which we scale to the instantaneous growth rate by choosing the function to be proportional to *N*(*t*):

$$\frac{dN\left(t\right)}{dt}=NG\left(\frac{N}{N_{T}}\right)+N\left(t\right)F\left(t\right).$$ (75)

If we introduce a new variable by the transformation:

$$U\left(t\right)=\ln\left[N/N_{T}\right]\;;\;N=N_{T}e^{U},$$

then by substituting the new variable into Eq. 75 we obtain:

$$\frac{dU\left(t\right)}{dt}=G\left(e^{U\left(t\right)}\right)+F\left(t\right),$$ (76)

which has the structure of a nonlinear Langevin equation with a deterministic forcing term $G\left(e^{U\left(t\right)}\right)$ and a random force *F*(*t*). Equation 76 may be solved quite generally by means of a finite difference scheme for the deterministic function and a random walk (RW) process for the random force. If the RW is sufficiently simple, then analytic forms for the probability that the population grows to a level *U*(*t*) in a time *t* may be obtained from the nonlinear Langevin equation. To determine the PDF centered on the deterministic growth we must make some assumptions about the RW process generating *F*(*t*), that is, about the statistical character of the fluctuating environment. If we assume the random force is generated by a memoryless Wiener process we have:

$$\left\langle F\left(t\right)F\left(t^{\prime }\right)\right\rangle =2D\delta \left(t-t^{\prime }\right).$$ (77)

This assumption enables us to construct the Fokker-Planck equation (FPE) for the PDF:

$$\frac{\partial P\left(u,t|u_{0}\right)}{\partial t}=-\frac{\partial }{\partial u}\left[G\left(e^{u}\right)P\left(u,t|u_{0}\right)\right]+D\frac{\partial^{2}P\left(u,t|u_{0}\right)}{\partial u^{2}}$$ (78)

where *P* (*u*, *t*|*u*_0_)*du* is the probability that the dynamic variable *U*(*t*) is in the phase space interval $\left(u+du,u\right)$ at time *t* given the initial value *U* (0) = *u*_0_.

The FPE may be put in a more recognizable form to physicists by ignoring the dependence on the initial condition and introducing the nonlinear transformation:

$$P\left(u,t\right)=\psi \left(u,t\right)\exp\left[\frac{1}{2D}\overset{u}{\underset{0}{\int }}G\left(e^{u^{\prime }}\right)du^{\prime }\right],$$ (79)

into the FPE to yield:

$$\frac{\partial \psi }{\partial \tau }=\frac{\partial^{2}\psi }{\partial U^{2}}-\left(\frac{\partial G}{\partial U}+G^{2}\right)\psi ,$$ (80)

where we have scaled the time *τ* = *Dt*/4 and the population u=2DU . Note that this last equation has the form of the Schrödinger equation in Quantum Mechanics and therefore makes available a vast literature on the solution to Eq. 80 for prescribed forms of *G* (*e* ^*u*^). The Verhulst growth law $\left[G\left(x\right)=1-x\right]$ is the analogue of the Morse potential in molecular physics and that of Gompertz corresponds to the harmonic oscillator. The solution to Eq.80 for these cases, among others, was explored and the equilibrium PDF for the Gompertz case was determined to be Gaussian and that for the Verhulst case to be Poisson (Goel et al., 1971).

#### Multiple Species

The single species equation of growth was intended to model all the stable environmental effects determining the growth law of a particular cell. We now wish to generalize this expression to include the interaction between multiple kinds of cells. We postulate that a single kind of cell grows in proportion to its instantaneous population with a nonlinear growth rate, which is coupled to all the other cell species in the tissue:

$$\frac{dN_{j}}{dt}=N_{j}G_{j}\left(N_{1},N_{2},N_{3}\right),$$ (81)

here cell type #1 is healthy, #2 is cancerous and #3 is dead. The function $G_{j}\left(N_{1},N_{2},N_{3}\right)$ is a normalized growth law for the *j*th cell type and an interaction function, i.e., the interaction with other cell types has been removed from the fluctuating force of the model just discussed and made explicit. We assume that the functions $\left\{G_{j}\right\}$ do not depend explicitly on the time, and that there exists a set of equilibrium populations $\left\{n_{j}\right\}$ such that *G*_*j*_ (*n*_1_, *n*_2_, *n*_3_) = 0 and none of the equilibrium populations vanish. Consequently in the vicinity of an equilibrium point the growth functions can be expressed as the Taylor series expansions:

$$G_{j}={\bar{G}}_{j}+\underset{k=1}{\overset{3}{\sum }}\left(N_{k}-n_{k}\right)\frac{\partial {\bar{G}}_{j}}{\partial N_{k}}+\underset{l,k=1}{\overset{3}{\sum }}\left(N_{k}-n_{k}\right)\left(N_{l}-n_{l}\right)\frac{\partial^{2}{\bar{G}}_{j}}{\partial N_{k}\partial N_{l}}+\cdot \cdot \cdot ,$$ (82)

where ${\bar{F}}_{j}\left({\bar{G}}_{j}=0\right)$ is the value of the function calculated at the equilibrium level. Substituting the Taylor series into the rate equation and defining the coefficients:

$$a_{k}^{j}\equiv \frac{\partial {\bar{G}}_{j}}{\partial N_{k}}\text{ ; }b_{kl}^{j}\equiv \frac{\partial^{2}{\bar{G}}_{j}}{\partial N_{k}\partial N_{l}},$$ (83)

gives rise to:

$$\frac{dN_{j}}{dt}=\underset{k=1}{\overset{3}{\sum }}a_{k}^{j}N_{j}\left(N_{k}-n_{k}\right)+\underset{l,k=1}{\overset{3}{\sum }}b_{kl}^{j}N_{j}\left(N_{k}-n_{k}\right)\left(N_{l}-n_{l}\right)+\;\cdot \cdot \cdot$$ (84)

Note that if one neglects the second order and higher terms in the deviation from equilibrium and introduces the constants:

$$\kappa_{j}=-\underset{k=1}{\overset{3}{\sum }}a_{k}^{j}n_{k}\text{ ; }a_{k}^{j}=a_{jk}/\beta_{j},$$

then Eq. 85 can be written:

$$\frac{dN_{j}}{dt}=\kappa_{j}N_{j}+\frac{1}{\beta_{j}}\overset{3}{\underset{k=1}{\sum }}a_{jk}N_{j}N_{k}$$ (85)

which is the form of the multi-species interaction modeled by Lotka and Volterra as well as other investigators. In the LV model the quadratic terms are interpreted as binary collisions between species *j* and *k* with *a*_*jk*_ being positive if *j* eats (kills) *k* and negative for the reverse. The 1/*β*_*j*_ represents exchange rates of the various species so that ${\left(\beta_{j}/\beta_{k}\right)}^{-1}$ is the ratio of *k*’s lost (or gained) to *j*’s gained (or lost). Volterra postulated that the coefficient *a*_*jk*_ is antisymmetric (*a*_*jk*_ = − *a*_*kj*_) as required for the eating (killing) order mentioned above, and showed the existence of a constant of the motion for the dynamic system. The Volterra model has, therefore, been shown to be a first approximation to any situation in which the growth rate of a variable is proportional to its instantaneous value when the population is small and in which a steady-state value exists when there are interactions with other species.

Montroll (1972) used the general growth function to extend the LV model to the new form:

$$\frac{dN_{j}}{dt}=\alpha_{j}N_{j}+\frac{1}{\beta_{j}}\overset{3}{\underset{k=1}{\sum }}a_{jk}N_{j}\frac{\left(N_{k}^{\mu }-1\right)}{\mu },$$ (86)

which becomes the Volterra system when *μ* = 1 and the linear growth rate is:

$$\alpha_{j}=\kappa_{j}+\frac{1}{\beta_{j}}\overset{3}{\underset{k=1}{\sum }}a_{jk}.$$

On the other hand, as *μ* → 0, after some algebra Eq. 86 becomes the linear interaction equation in terms of the transformed variable *U*_*j*_ =  ln (*N*_*j*_/*n*_*j*_):

$$\frac{dU_{j}}{dt}=\frac{1}{\beta_{j}}\overset{3}{\underset{k=1}{\sum }}a_{jk}U_{k},$$ (87)

which may be solved by usual methods for linear IREs.

#### Fisher-Like Equation

We begin the discussion on the effects of diffusion on the growth of a species with a brief review of the classic problem in genetics developed by Fisher (1937). He was interested in the propagation of a virile mutant in a population living in a linear habitat, an example of which would be a species living along a seacoast. He developed his dynamic equations with a RW argument involving finite difference equations defined on a lattice. We skip to the continuous limit of his RW argument and write:

$$\frac{\partial }{\partial t}p\left(x,t\right)=D\frac{\partial^{2}}{\partial x^{2}}p\left(x,t\right)+\kappa p\left(x,t\right)\left[1-p\left(x,t\right)\right],$$ (88)

which has both the features of self-limited (saturated) growth and diffusion. Here, *p* (*x*, *t*)*dx* is the relative frequency of the mutant strain in the population at the position *x* and time *t*, and *κ* is the advantage of the mutant strain under conditions of random mating.

Skellam (1951) considered a linear RW in space consisting of computation cells containing a growing population and obtained an equation of the same form as Fisher’s, as well as others. A slightly more general form for this diffusion equation was obtained for the population *ρ*(*x*, *t*) = *N*_*T*_ *p* (*x*, *t*) with a finite saturation level *N*_*T*_, but that equation has proven to be no more amenable to general closed form solution than Fisher’s original equation. However, if we can obtain analytic solutions in the region near saturation and another exact solution in a region far from saturation *N*_*T*_ ≫ *ρ* they can be used to bracket the exact solution to Fisher’s equation, if it exists. Moreover if the solution to Fisher’s equation is continuous, then it must join the two asymptotic solutions at the extremes of population growth.

The direct solution of Eq. 88 is extremely difficult to obtain due to the nonlinear structure of the equation. Fisher (Fisher, 1937) and Skellam (Skellam, 1952) obtained numerical solutions assuming the form of a diffusion wave *p* (*x*, *t*) = *p* (*x* − *vt*). A more general analytic solution may be obtained in a restricted region, say near saturation. Let us consider the expansion:

$$\frac{\rho \left(x,t\right)}{N_{T}}=e^{\ln\left[\rho \left(x,t\right)/N_{T}\right]}\approx 1+\ln\left[\rho \left(x,t\right)/N_{T}\right]+\cdot \cdot \cdot ,$$ (89)

the first two terms of which give a good representation of the ratio $\rho \left(x,t\right)/N_{T}$ in the region near saturation. Substituting this expansion into Eq. 88 and introducing the new variable $u\left(x,t\right)=\ln\left[\rho \left(x,t\right)/N_{T}\right]$ into the resulting equation yields:

$$\frac{\partial }{\partial t}u\left(x,t\right)=D\frac{\partial^{2}}{\partial x^{2}}u\left(x,t\right)-\kappa u\left(x,t\right),$$ (90)

It is clear that this equation provides a solution to the Fisher equation near saturation. To obtain a closed form solution, we strain Fisher’s example of a linear habitat and require that the population lie along the perimeter *L* of an island such that *u* (*x* + *L*, *t*) = *u* (*x*, *t*). With this assumption of periodicity it is straightforward to show that the solution to Eq. 90 for an initial distribution *u*_0_(*x*) = *u*(*x*, *t* = 0) is:

$$u\left(x,t\right)=e^{-\kappa t}\underset{-\infty }{\overset{\infty }{\int }}G\left(x-x^{\prime },t\right)u_{0}\left(x^{\prime }\right)dx^{\prime },$$ (91)

where G(x,t) is the Gauss distribution solution of the homogeneous diffusion equation and here plays the role of a Greens function.

If we transfer Eq. 90 back to the population variable *ρ*, we obtain after a little algebra:

$$\frac{\partial \rho }{\partial t}=D\left[\frac{\partial^{2}\rho }{\partial x^{2}}-\frac{1}{\rho }{\left(\frac{\partial \rho }{\partial x}\right)}^{2}\right]-\kappa \rho \ln\left(\frac{\rho }{N_{T}}\right),$$ (92)

as the approximate form of the Fisher equation in the region near saturation, whose analytic solution is:

$$\rho \left(x,t\right)=\theta \exp\left[e^{-\kappa t}\underset{-\infty }{\overset{\infty }{\int }}G\left(x-x^{\prime },t\right)\ln\left[\frac{\rho_{0}\left(x^{\prime }\right)}{N_{T}}\right]dx^{\prime }\right].$$ (93)

A similar solution may be found in the region far from saturation *ρ* ≪ *N*_*T*_, that being the solution to the equation:

$$\frac{\partial \rho \left(x,t\right)}{\partial t}=D\frac{\partial^{2}\rho \left(x,t\right)}{\partial x^{2}}+\kappa \rho \left(x,t\right).$$ (94)

Thus, we are able to bracket the exact solution the Fisher equation even though we do know its analytic form.

### Comments on Fractional Diffusion

From previous sections we know that we can introduce memory into the population dynamics through a subordination process and thereby obtain a fractional time diffusion equation (FTDE) of the form:

$$\partial_{t}^{\alpha }\left[\rho \left(x,t\right)\right]=D\frac{\partial^{2}\rho \left(x,t\right)}{\partial x^{2}},$$ (95)

which is expressed in terms of the Fourier transform of the population $\tilde{\rho }\left(k,t\right)$ as:

$$\partial_{t}^{\alpha }\left[\tilde{\rho }\left(k,t\right)\right]=-Dk^{2}\tilde{\rho }\left(k,t\right).$$ (96)

Alternatively, the FTDE can be expressed in terms of the Laplace transform of the population $\hat{\rho }\left(x,s\right)$ as:

$$s^{\alpha }\hat{\rho }\left(x,s\right)-s^{\alpha -1}\rho_{0}\left(x\right)=D\frac{\partial^{2}\hat{\rho }\left(x,s\right)}{\partial x^{2}},$$ (97)

where the fractional time derivative is of the Caputo type. The most efficient way to solve the FTDE is to invert the joint Fourier-Laplace transform of the population ρ*(*k*, *s*) which assumes the form:

$$\rho^{*}\left(k,s\right)=\frac{s^{\alpha -1}{\tilde{\rho }}_{0}\left(k\right)}{s^{\alpha }+Dk^{2}}.$$ (98)

For a point source initial condition $\rho_{0}\left(x\right)=\delta \left(x\right)$ we have ${\tilde{\rho }}_{0}\left(k\right)=1$ and the inversion of this equation yields:

$$\rho \left(x,t\right)={\mathrm{FT}}^{-1}\left\{E_{\alpha }\left(-Dk^{2}t^{\alpha }\right);x\right\},$$ (99)

with the solution to the FTDE in terms of the inverse Fourier transform of the MLF.

We also know from previous discussions that spatial heterogeneity can be introduced into the population dynamics through the network effect and thereby obtain a fractional space diffusion equation (FSDE) of the form:

$$\frac{\partial \rho \left(x,t\right)}{\partial t}=D\partial_{\left|x\right|}^{\beta }\left[\rho \left(x,t\right)\right],$$ (100)

where $\partial_{\left|x\right|}^{\beta }\left[\cdot \right]$ is the Riesz-Feller fractional derivative (West, 2016). The solution to this FSDE can be expressed in terms of the inverse of the Fourier-Laplace transform:

$$\rho^{*}\left(k,s\right)=\frac{{\tilde{\rho }}_{0}\left(k\right)}{s+D{\left|k\right|}^{\beta }},$$ (101)

where the Fourier transform of the Riesz-Feller fractional derivative in one spatial dimension is $-{\left|k\right|}^{\beta }$ . Inverting Eq. 101 for the same point source initial condition used previously gives us:

$$\rho \left(x,t\right)=\overset{\infty }{\underset{-\infty }{\int }}\frac{dk}{2\pi }e^{ikx}e^{-D{\left|k\right|}^{\beta }t},$$ (102)

which is a Lévy stable PDF. Note that when *α* = 1 the MLF in Eq. 99 becomes an exponential and the PDF reduces to a Gauss PDF which is a *β* = 2 Lévy stable form (West, 2017).

It is worth mentioning that the FTDE is a version of (Evangelista and Lenzi, 2018):

$$\begin{array}{ccc} \partial_{t}^{\alpha }\left[\rho \left(x,t\right)\right] & = & D\frac{\partial^{2}\rho \left(x,t\right)}{\partial x^{2}}-\frac{\partial }{\partial x}\left[F\left(x,t\right)\rho \left(x,t\right)\right], \end{array}$$ (103)

$$\begin{array}{ccc} & = & \frac{\partial J\left(x,t\right)}{\partial x}, \end{array}$$ (104)

where is the influence of the environment is modeled as an external force *F* (*x*, *t*) and *J* (*x*, *t*) is the population current density. A exhaustive mathematical discussion of fractional anomalous diffusion is given by Evangelista and Lenzi in their remarkably timely book (Evangelista and Lenzi, 2018).

## Closing Thoughts

In this all too brief introduction to the growing area of application of the fractional calculus to MO we have covered many mathematical concepts, each new wrinkle capturing a different nuance in the complexity of biomedical phenomena. Rather than attempting a detailed summary of what has been presented herein, we instead identify and articulate a number of general results. We will then attempt to put these remarks into a larger context and anticipate some of the future research directions that may facilitate the modeling of biomedically complex phenomena and pathologies.

We begin by identifying the most important points covered in this essay:

1) The simple analytic functions of the IC have been shown in the prequel to be insufficient to describe the time dependence of most physiology networks. The notion of fractality was introduced to capture the true complexity of such biomedical networks through fractal geometry, fractal statistics and fractal dynamics.
2) A fractal function diverges when an integer-order derivative is taken, so that such a fractal function cannot be the solution to a Newtonian equation of motion. However, when a fractional-order derivative of a fractal function is taken it results in a new fractal function. Consequently, a time-dependent fractal process can have an equation of motion that is a FDE.
3) The Network Effect is the influence exerted by a complex dynamic network on each member of the network. When the network dynamics is a member of the Ising universality class the interconnected set of IDEs for the probability of an individual being in one of two states during its nonlinear interaction with the other members of the network can be replaced by an equivalent linear FDE.
4) One of the simplest FDEs has a built-in memory resulting from the hidden interaction of the observable with its environment, which is manifest in the non-integer order of the time derivative, as in the network effect. Examples include the deviation of experimental results of Newton’s Law of Cooling from predictions using an IRE and the dynamics of the very early time description of Brownian motion also using an IRE.
5) Another simple FDE has a built-in non-locality in space and is the FSDE. The solution to this fractional diffusion equation in space is a Lévy PDF, whose index is given by the order of the spatial fractional derivative. Yet another fractional diffusion equation differs in having a built-in memory and is the FTDE. The solution to this fractional diffusion equation in time is expressed in terms of the inverse Fourier transform of a MLF.
6) The solution to a linear FRE is a MLF for *α* < 1 and becomes an exponential function for *α* = 1. The MLF is the workhorse of the FC just as the exponential is for the IC.
7) A truly complex stochastic dynamic process can have more than one fractal dimension. A multifractal process is characterized by a uni-modal spectrum $f\left(h\right)$ peaked at the value of the Hurst exponent *h* = *H*.
8) The time-dependent fractional-order index $\alpha \left(t\right)$ specifies a distributed-order fractional operator. As a sufficiently rich complex process evolves over time its fractal dimension changes to explore the full range of dimensionality $0<\alpha \left(t\right)\leq 1$ through the multifractal spectrum $f\left(\alpha \right)$ .

The short term goal of this essay has been, in part, to describe how the growth of natural biological phenomena differs from the growth of physical phenomena. We explored this by showing how to incorporate memory effects into the growth process of biological cells by replacing IREs with FREs. The replacement of integer-order with fractional-order derivatives in time required a brief foray into the solving of the FREs that describe the growth of cells over time, including the saturation of growth using Verhulst (logistic) and Gompertz models. Such descriptions are important in order to understand the multiscale processes that emerge when tissues are electrically stimulated or mechanically stressed (Magin, 2010), as well as being pathologically disrupted.

We close these remarks by emphasizing the nexus between distributed-order differentiation and multifractality. The invariance of scale is a property relating time series across multiple scales and has provided a new perspective regarding medicine, physiological phenomena and their associated control systems. The historical engineering paradigm of ‘signal-plus-noise’ was first replaced by a model of biological time series that had fractal statistics. This however was also shown to be too restrictive when a number of physiological signals were found to be characterized by more than one scaling parameter and therefore to belong to a class of complex processes known as multifractals. Such multifractal time series appear in the rich healthy variability of both human gait and heart rate (Ivanov et al., 1999; West, 2006; Bogdan et al. 2020).

We use blood flow within the brain as an exemplar of how the multifractal character of health can be described and subsequently explained using the FC. West et al., 2003b) demonstrated that the scaling properties of the time series associated with cerebral blood flow (CBF) significantly differs between that of normal healthy individuals and migraineurs. The CBF time series discussed here is typical of physiologic signals generated by complex self-regulated networks that handle inputs having a broad range of scales. An indirect way of measuring CBF is by monitoring the blood flowing into the brain through the middle cerebral artery. This can be accomplished using an instrument that operates like a radar gun, but instead of scattering electromagnetic waves from your car back to the gun to determine your speed, it scatters acoustic (sound) waves from the fluctuations in the blood back to the gun to determine the flow velocity. The instrument is a transcranial Doppler ultrasonograph and provides a high resolution measurement of middle cerebral artery flow velocity. We look for the signature of the migraine pathology in the scaling properties of the human middle cerebral artery CBF velocity time series.

**FIGURE 11:**
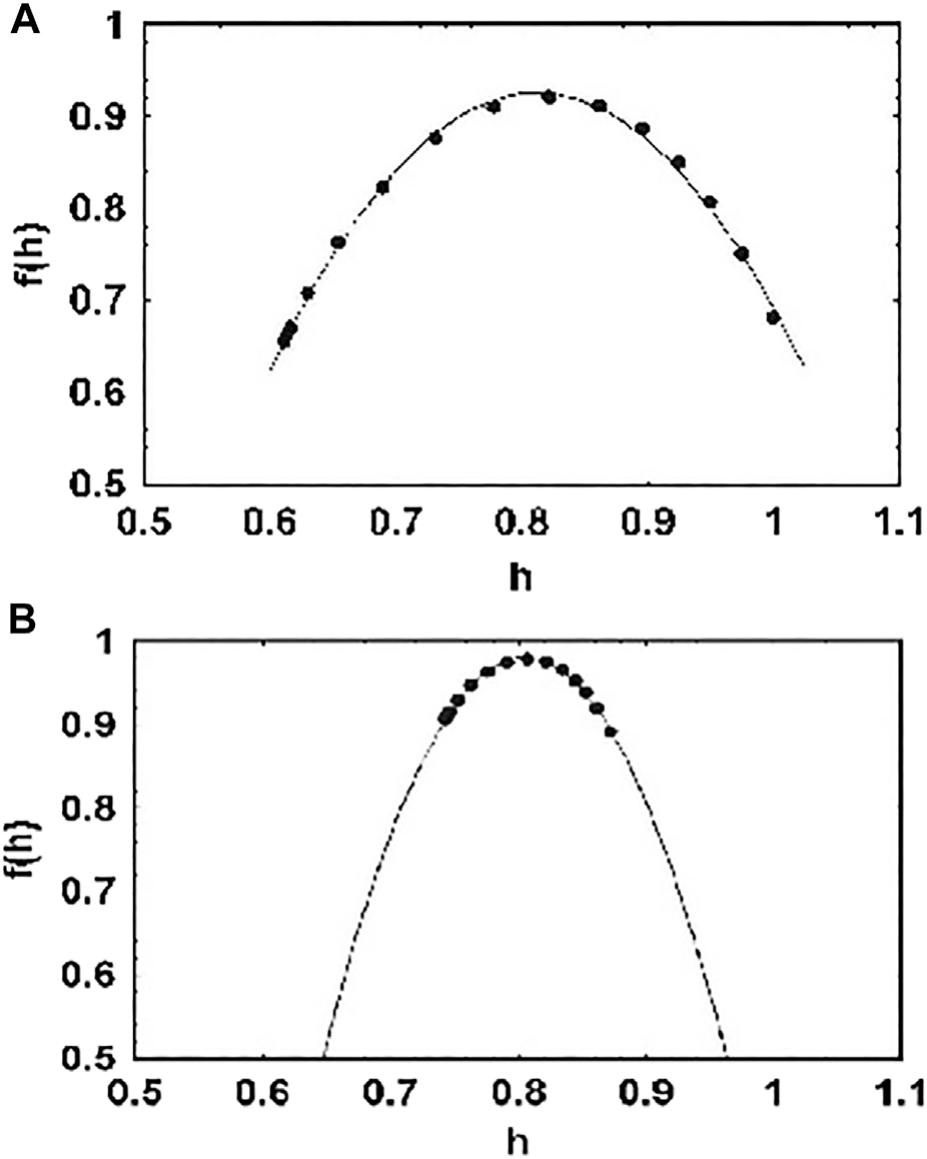
The average multifracal spectrum for middle CBF time series is depicted by *f(h)*. **(A)** The spectrum is the average of 10 time series measurements of five healthy subjects (filled circles). The solid line is the best least-squares fit of the parameters to the predicted spectrum. **(B)** The spectrum is the average of 14 time series measurements of eight migraineurs (filled circles). The solid curve is the best least-squares fit to a predicted spectrum. From (West et al., 2003b) with permission.

The properties of monofractals are determined by the local scaling exponent, but as mentioned multifractals are made up of many interwoven subsets with different local scaling exponents. The statistical properties of these subsets are characterized by the spectral distribution of fractal dimensions *f(h)* as depicted in Figure 11. In this figure we compare the multifractal spectrum for the middle CBF velocity time series for a healthy group of five subjects and a group of eight migraineurs.

A significant change in the multifractal properties of the middle CBF velocity time series from the control group to that of the migraineurs. is apparent. Namely, the width the multifractal spectrum of the local scaling exponent is vastly constricted, being reduced by a factor of three from 0.038 for the control group to 0.013 for the migraineurs. The multifractal spectrum for migraineurs is centered at 0.81, the same as that of the control group, so the average scaling behavior would appear to be the same. However, the narrowing of the fractal dimension spectrum suggests that the underlying process has lost its flexibility. The advantage of multifractal processes is that they are highly adaptive, so that in this case the brain of a healthy individual adapts to the multifractality of the interbeat interval time series of the heart. We see that the disease, in this case migraine, may be associated with the loss of complexity (Goldberger et al., 1990), due to the narrowing of the spectral width, and consequently the loss of adaptability, thereby suppressing the normal healthy multifractality of CBF time series.

The experimental evidence presented in the prequel supports the interpretation that the greater the complexity of the physiologic time series, as measured by the width of the multifractal spectrum, the healthier the physiological network. In addition, theory (West and Grigolini, 2021) suggests that the information transfer between two coupled networks is from the network with the wider spectrum (greater complexity) to that with the narrower spectrum (lesser complexity). We hypothesize that the multifractal dynamics of oncological processes may be well represented by distributed-order FDEs that captures the loss of complexity in the transition from healthy multifractal physiologic processes with a substantial spectral width to a pathological process with a significantly narrower spectral width. This hypothesis will be the focus of the next essay in this sequence.

To end this essay on a positive note, we briefly mention a number of the topic areas suggested by thoughtful reviewers of the manuscript, which although relevant to the theme of this essay we lacked the skill to incorporate them into the present text. There have been numerous efforts dealing with observability and controllability of physiological networks while considering the FD observed in medicine, see for example, (Bogdan, 2019; Kyriakis et al., 2020). Another is to use what we know concerning the information exchange between complex networks (West et al., 2008; West and Grigolini, 2021) to implement the FC for reducing the risk of closed loop control of blood glucose in artificial pancreas (Ghorbani and Bogdan, 2014), but also in optimal control theory where it may lead to a new branch of control techniques such as time-dependent fractal optimal control.

The acknowledgement of this new perspective is nowhere more evident than in the timely launching of a journal that recognizes the emergening field of Network Physiology (Ivanov et al., 2016; Ivanov, 2021). I wholeheartedly endorse this new journal with but a single reservation, Ivanov’s reference to Network Physiology as being ‘multi-disciplinary’ (Ivanov, 2021). I much prefer the less restrictive term ‘*trans*-disciplinary’, in large part because with the future application of the FC to Network Medicine as well as to Network Physiology will itself generate disciplines that will not fit into our present day taxonomy of scientific disciplines.

## Data Availability

The original contributions presented in the study are included in the article/Supplementary Material, further inquiries can be directed to the corresponding author.
